# PMeS: Prediction of Methylation Sites Based on Enhanced Feature Encoding Scheme

**DOI:** 10.1371/journal.pone.0038772

**Published:** 2012-06-15

**Authors:** Shao-Ping Shi, Jian-Ding Qiu, Xing-Yu Sun, Sheng-Bao Suo, Shu-Yun Huang, Ru-Ping Liang

**Affiliations:** 1 Department of Chemistry, Nanchang University, Nanchang, People’s Republic of China; 2 Department of Mathematics, Nanchang University, Nanchang, People’s Republic of China; University College Dublin, Ireland

## Abstract

Protein methylation is predominantly found on lysine and arginine residues, and carries many important biological functions, including gene regulation and signal transduction. Given their important involvement in gene expression, protein methylation and their regulatory enzymes are implicated in a variety of human disease states such as cancer, coronary heart disease and neurodegenerative disorders. Thus, identification of methylation sites can be very helpful for the drug designs of various related diseases. In this study, we developed a method called PMeS to improve the prediction of protein methylation sites based on an enhanced feature encoding scheme and support vector machine. The enhanced feature encoding scheme was composed of the sparse property coding, normalized van der Waals volume, position weight amino acid composition and accessible surface area. The PMeS achieved a promising performance with a sensitivity of 92.45%, a specificity of 93.18%, an accuracy of 92.82% and a Matthew’s correlation coefficient of 85.69% for arginine as well as a sensitivity of 84.38%, a specificity of 93.94%, an accuracy of 89.16% and a Matthew’s correlation coefficient of 78.68% for lysine in 10-fold cross validation. Compared with other existing methods, the PMeS provides better predictive performance and greater robustness. It can be anticipated that the PMeS might be useful to guide future experiments needed to identify potential methylation sites in proteins of interest. The online service is available at http://bioinfo.ncu.edu.cn/inquiries_PMeS.aspx.

## Introduction

Protein methylation, which was discovered more than 40 years ago [1], is an important and reversible protein post-translational modification (PTM). This PTM includes N-methylation [2], [3], [4] of either the backbone or side-chain of arginine, lysine, histidine, proline, alanine and asparagine, O-methylation [5] of either internal carboxyl groups of glutamate or isoaspartate residues and COOH-terminal lipidated cysteine residues, and S-methylation [6] of either cysteine or methionine residues. Among them, arginine and lysine are the most frequently methylated residues. Arginine methylation is catalyzed by a family of enzymes called protein arginine methyltransferases (PRMTs) [7]. PRMTs are classified into two groups, type I PRMTs catalyze the formation of N^G^-monomethylarginine (MMA) and asymmetric ω-N^G^, N^G^-dimethylarginine (aDMA), type II enzymes form MMA and symmetric ω-N^G^, N′^G^-dimethylarginine (sDMA) [8]. Similarly, lysine methylation involves the addition of one to three methyl groups on the amino acid’s ε-amine group, to form mono-, di- or tri-methyllysine by lysine methyltransferases (KMTs) [2]. Lysine specific demethylases (KDMs) work in coordination with histone lysine methylases to maintain global histone methylation patterns [9].

It has now been shown that protein arginine methylation has an important role in gene regulation and signal transduction, and lysine methylation is correlated with either gene activation or repression depending on the site and degree of methylation [10]. Given their important involvement in gene regulation, arginine methylation, lysine methylation and their regulatory enzymes are implicated in a variety of human disease states such as cancer [9], [11], coronary heart disease [12], multiple sclerosis [13], rheumatoid arthritis [14] and neurodegenerative disorders [15]. Thus, understanding the mechanisms governing these basic epigenetic phenomena will surely represent a very attractive target for drug discovery to prevent the onset of various related diseases. Furthermore, identification of protein methylation sites is of fundamental importance to understand the methylation dynamics and molecular mechanism. Unfortunately, it is often laborious, time intensive and expensive to determine protein methylation sites using conventional experiments including methylation-specific antibodies, Chip-Chip and mass spectrometry [16]–[18]. Therefore, a robust computational prediction tool is desirable to reduce the number of experiments needed to identify potential methylation sites in proteins of interest.

Actually, several computational methods have been developed to handle these methylation sites prediction problems from primary protein sequences. Plewczynski *et al.* [19] designed the first methylation sites predictor within their AutoMotif Server using regular expression technique. Subsequently, Daily *et al.* [20] developed a method for arginine and lysine methylation prediction, using support vector machine (SVM) based on the hypothesis that PTMs preferentially occur in intrinsically disordered regions. Chen *et al.* [21] built a web server MeMo for identifying methylation sites by utilizing orthogonal binary coding scheme to represent protein sequence fragment. Further, Shao *et* *al.* [22] combined Bi-profile Bayes feature extraction with SVM to predict arginine and lysine methylation. MASA was constructed by Shien *et al.* [23] for methylation sites prediction, where considered both sequence information and structural characteristics such as accessible surface area (ASA) and secondary structure of residues surrounding methylation sites. Recently, Hu *et al.* [24] presented a method for predicting protein methylarginine and methyllysine based on multi-sequence features and nearest neighbor algorithm.

However, most existing prediction methods applied orthogonal encoding scheme to characterize protein sequence information. The orthogonal encoding uses a 20 dimensional vector of binary values 0 or 1 to represent each residue. Each bit in this vector means the occurrence of one kind of amino acid. Thus, there is one 1 and nineteen 0 in each vector. It is obvious that orthogonal representation doesn’t contain preferences on amino acids or position information and physicochemical properties of residues. Additionally, the highest prediction sensitivity was 82.1% for methylarginine [23], only 79.73% for methyllysine among the existing methods [24]. Hence it has become a crucial issue to improve the quality of predicting protein methylation sites by selecting more informative feature descriptors.

In view of this, a novel approach called PMeS was developed to identify methylation sites based on an enhanced feature encoding scheme for extracting the most informative amino acids features. Here, the enhanced feature encoding scheme was composed of sparse property coding (SPC), normalized van der Waals volume (VDWV), position weight amino acid composition (PWAA) and solvent accessible surface area (ASA). SPC and VDWV were utilized to characterize protein sequence information and physicochemical properties of amino acids surrounding methylation sites. PWAA and ASA were applied to represent sequence-order information and structural characteristic around methylation sites, respectively. Our current work contained the following contents: (1) four types of features and feature analysis were considered; (2) SVM was employed to deal with the problem of binary classification; (3) ten-fold cross-validation method was chosen to evaluate the performance of SVM classifier; (4) the effect of window length was discussed; (5) the ratio of positive to negative samples was investigated; (6) the robustness of PMeS was considered; and (7) the predictive performance of PMeS was compared with that of the existing models.

**Figure 1:**
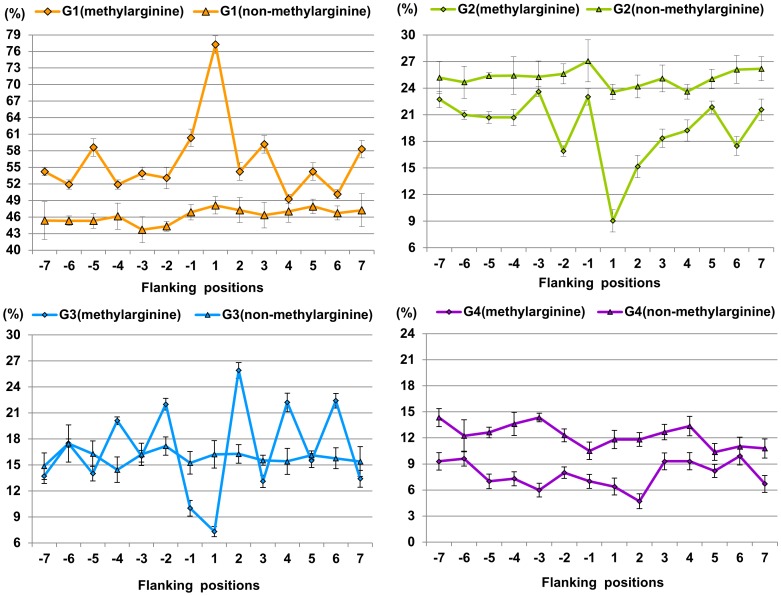
The distribution of physicochemical properties of residues around methylarginine and non-methylarginine. G1 is hydrophobic residue, G2 is polar residue, G3 is positively charged residue, and G4 is negatively charged residue.

## Materials and Methods

### Data Collection

All training data were extracted from UniProtKB/Swiss-Prot database (version 2011_05, www.uniprot.org) and PhosphoSitePlus (2011_05, www.phosphosite.org). Firstly, we obtained 98 proteins covering 246 experimental methylarginine sites by searching information containing “Omega-N-methylarginine”, “symmetric dimethylarginine” and “asymmetric dimethylarginine”, and 137 proteins covering 367 experimental methyllysine sites through the keywords “N6, N6, N6-trimethyllysine”, “N6, N6-dimethyllysine” and “N6-methyllysine” from UniProtKB/Swiss-Prot database (see Tables S1 and S2). PhosphoSitePlus is an online systems biology resource providing an extensive, manually curated phosphorylation site database and other commonly studied PTMs including acetylation, methylation, ubiquitination, and O-glycosylation. We obtained 68 non-redundant proteins covering 155 experimental methylarginine sites and 78 non-redundant proteins covering 147 experimental methyllysine sites from PhosphoSitePlus (see Tables S3 and S4). However, the dataset may contain several high sequence identity proteins. To avoid such overestimation of predictive performance, we clustered the protein sequences with a threshold of 40% identity by CD-HIT program [25] to remove the highly homologous sequences.

Secondly, the sliding window strategy was utilized to extract positive and negative data from protein sequences as training data, which were represented by peptide sequences with arginine and lysine symmetrically surrounded by flanking residues. Experimentally validated methylarginine and methyllysine were defined as positive datasets, excluding those annotated by “potential”, “probable” or “by similarity” in the description field. Negative datasets included all arginines and lysines that were not marked by any methylation information on the same proteins. Although not all of these sites are necessarily true negatives, it is reasonable to believe that a large majority of them are [26]. Moreover, the redundancy reducing process was also carried out on training data. For example, for two methylated arginine peptide sequences with 100% identity, when the methylarginine sites in the two proteins were in the same positions, only one was kept. After strictly following the above procedures, we attained 355 high quality positive sites and 3960 negative sites for methylarginine, and 322 positive sites and 4126 negative sites for methyllysine. Here, the feasible window size for both arginine and lysine was 15 after several trials of 9, 11, 13, 15, 17 and 19.

Finally, to ensure unbiased and objective results, five negative training sets were obtained by randomly extracting from the negative datasets. The average predictive performance obtained using the five sets of training data was calculated by the following cross-validation.

**Figure 2:**
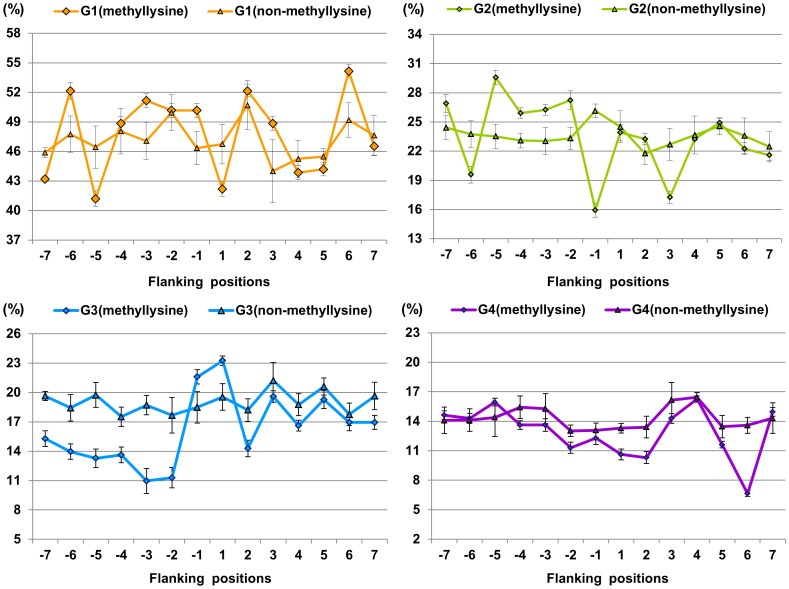
The distribution of physicochemical properties of residues around methyllysine and non-methyllysine. G1 is hydrophobic residue, G2 is polar residue, G3 is positively charged residue, and G4 is negatively charged residue.

### The Enhanced Feature Encoding Scheme

#### Sparse property coding

The specificity and diversity of protein structure and function are largely attributed to the composition of various properties of each of the 20 amino acids [27]. Physicochemical encoding is particularly suited for peptides since it exploits the fixed length of the sequence [28]. Peptide sequences have been coded using physicochemical properties in three ways: sparse property coding, continuous property coding and property projection coding [29]. Methylation on lysine and arginine residues does not alter their charge, but it does increase their hydrophobicity [30], [31]. Thus, we adopted a sparse property coding based on the hydrophobicity and charged character of amino acid residue. The sparse property coding (SPC) divided the 20 amino acid residues into four different groups according to their hydrophobicity and charged character: the hydrophobic group *G_1_* = {*A,F,G,I,L,M,P,V,W*}, the polar group *G_2_* = {*C,N,Q,S,T,Y*}, the positively charged group *G_3_* = {*H,K,R*} and the negatively charged group *G_4_* = {*D,E*} [32]. Then each amino acid residue
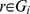
was encoded as follows:
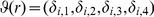
(1)where 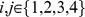 and 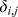 is the Kronecker delta symbol. Consequently, a peptide sequence *p* with sliding window size *N* can be mapped to a *4N*-dimension vector
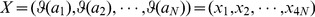
(2)within the feature space by concatenating the encoded amino acids, where 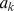 is the kth position residue in peptide sequence p.

The SPC reflects the distribution of residues with the same unique characteristic and portrays the essence of protein sequence. It can effectively overcome the defect of orthogonal encoding which doesn’t contain physicochemical properties of amino acids. On the other hand, the SPC reduces the dimension of the input space, so the computational complexity is largely decreased.

#### Van der Waals volume (VDWV)

Van der Waals volume of side groups is a determinant for binding sites [33]. Therefore, we took into account the normalized van der Waals volume (VDWV) of the amino acid side chain as a feature to code the peptides. The normalized van der Waals volume of 20 kinds of amino acids is presented in Supplementary Table S5 [34].

**Figure 3:**
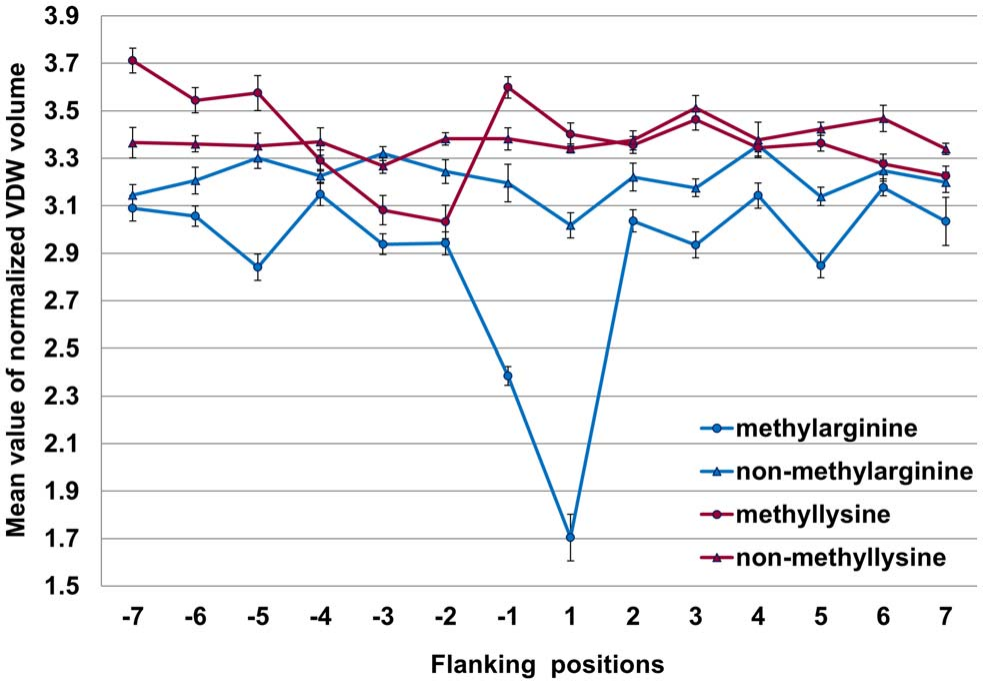
The mean value of normalized van der Waals volume (VDWV) of residues around methylation sites and non-methylation sites.

#### Position weight amino acid composition

To avoid losing the sequence-order information, we presented position weight amino acids composition (PWAA) to extract the sequence position information of amino acid residues around the methylation sites and non-methylation sites. Given an amino acid residue *a_i_* (*i* = 1,2,…,20), we can express the position information of amino acid *a_i_* in the protein sequence fragment *p* with 2*L* +1 amino acids by following formula:
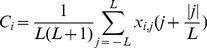
(3)where *L* denotes the number of upstream residues or downstream residues from the central site in the protein sequence fragment *p*, 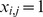 if *a_i_* is the *j*th position residue in protein sequence fragment *p*, otherwise 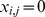. In general, the closer residue *a_i_* is to the central site (0 position), the absolute value of 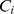 is smaller. Finally, a protein sequence fragment *p* is defined as 20 dimension feature vectors.
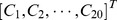
(4)


#### Solvent accessible surface area

A side-chain of amino acid that undergoes post-translational modification (PTM) prefers to be accessible on the surface of a protein [35]. Pang *et al.* [35] investigated the structural environment of 8378 incidences of 44 types of post-translational modifications (PTMs). It has been observed that protein methylation prefers to occur in regions that are intrinsically disorder and easily accessible. Therefore, the solvent accessibility of amino acid residues surrounding the methylation sites may be adapted to evaluate the classifying performance when distinguishes between the methylation site and non-methylation sites.

Most of the experimental methylation proteins do not have corresponding protein tertiary structures in the protein data bank (PDB). Consequently, we used RVP-Net [36], [37] to calculate the solvent accessible surface area (ASA) for each residue of a protein sequence. RVP-net applied a neural network to predict real value of ASA of residues based on neighborhood information, with 18.0–19.5% mean absolute error, defined as per residue absolute difference between the predicted and experimental values of relative ASA [36]. The computed ASA value was the percentage of the solvent-accessible area of each amino acid on the protein sequence. The ASA values of amino acids surrounding the methylation site were extracted and normalized.

### Support Vector Machine

SVM is a supervised learning method for classification and regression designed by Vapnik [38]. The principle of the SVM method is to transform the samples into a high dimension Hilbert space and seek an optimal separating hyperplane which maximizes the margin in feature space. SVM has shown successful ability to classify complex data sets without over-fitting issues, thus it’s considered as a machine learning tool for methylation prediction. For actual implementation we used the LIBSVM package (version 3.0) [39]. Here, a radial basis function was chosen as the kernel function, the penalty parameter and the kernel width parameter were tuned based on the training set using the grid search strategy in LIBSVM.

### Evaluation Methods

Ten-fold cross-validation was applied to evaluate the powers of the prediction method proposed in this study. The training data are divided into 10 groups by splitting each dataset into 10 approximately equal-sized subgroups. Then 9 subgroups are merged into a training data set while the remnant subgroup is taken as a testing data set. This process is repeated 10 times and the average performance of 10-fold cross-validation is used to estimate the performance. We adopted four major parameters for performance assessment: sensitivity (*Sn*), specificity (*Sp*), accuracy (*Acc*) and Matthews Correlation Coefficient (*MCC*). All of the above measurements are defined as follows:
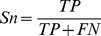
(5)

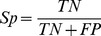
(6)

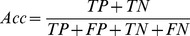
(7)

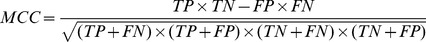
(8)where *TP,TN,FP,FN* denote the number of true positives, true negatives, false positives and false negatives, respectively. Sensitivity and specificity illustrate the correct prediction ratios of positive (methylation) samples and negative (non-methylation) samples respectively, while accuracy represents the correct ratio among both positive and negative data sets. The *MCC* takes into account true and false positives and negatives, and it is generally regarded as a balanced measure which can be used even if the classes are of very different sizes, for these reasons the *MCC* is more reliable than the accuracy. The value of *MCC* ranges from −1 to 1, and a larger *MCC* stands for better prediction performance.

**Figure 4:**
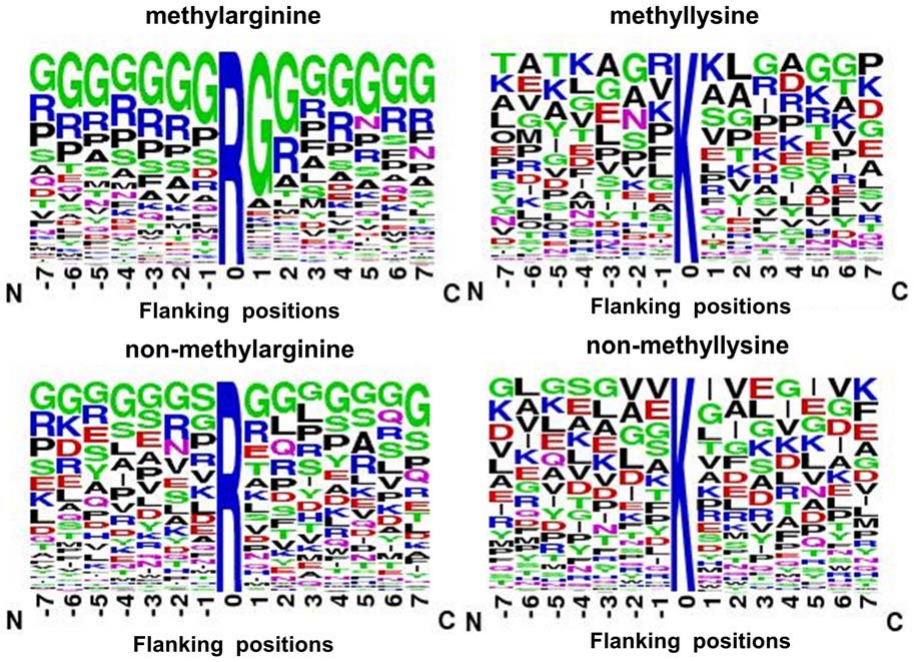
Sequence logo plots of methylation sites and non-methylation sites represent normalized amino acid frequencies for ±7 amino acids.

**Figure 5:**
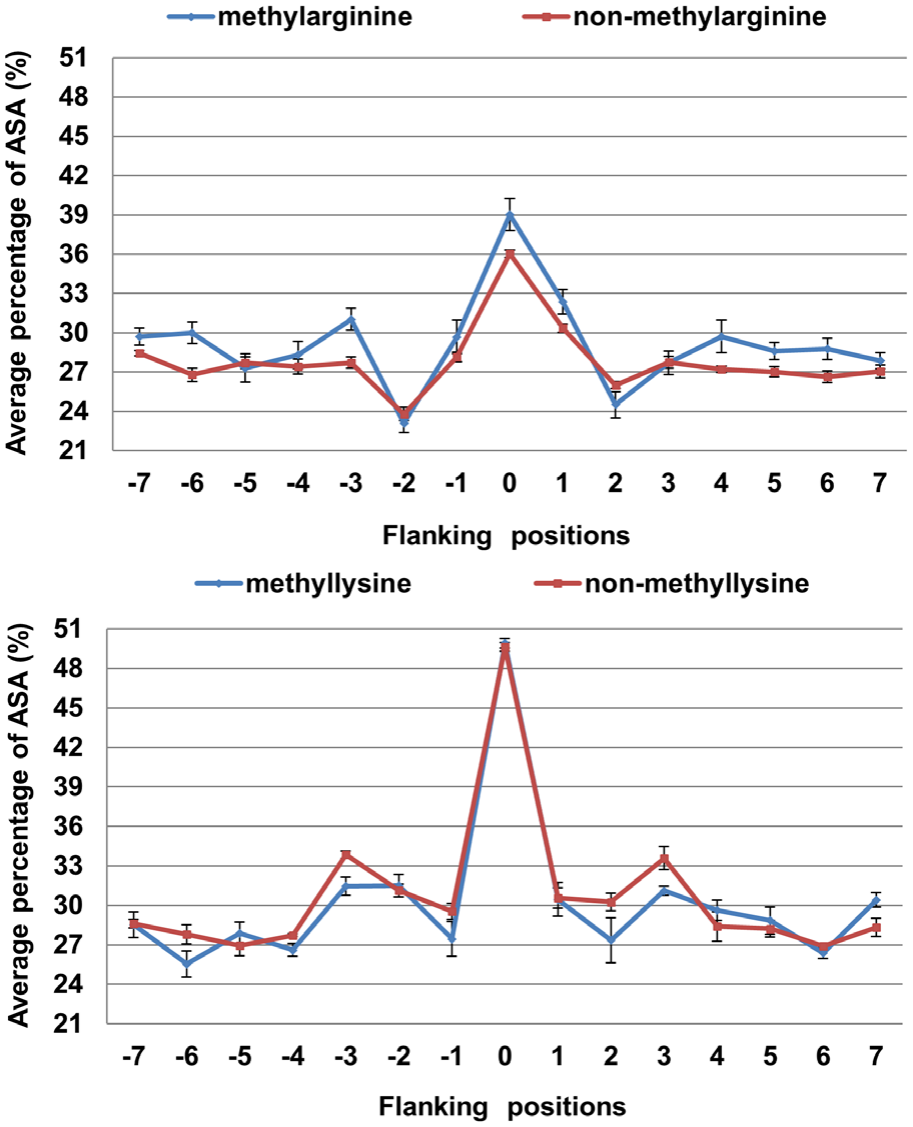
The average accessible surface area (ASA) of residues around methylation sites and non-methylation sites.

## Results and Discussion

### Investigation of Different Features

As described in the Materials and Methods section, the enhanced feature encoding scheme included four types of features: sparse property coding (SPC), normalized van der Waals volume (VDWV), position weight amino acids composition (PWAA) and solvent accessible surface area (ASA). Here we constructed ten prediction models composed by SPC, VDWV, PWAA and ASA to investigate the influences of different features.

### SPC Feature Analysis

As mentioned above, the SPC feature is mainly based on the hydrophobicity and charged character of amino acid residue. To determine whether methylation and non-methylation sites have distinct physiochemical properties, we calculated statistically significant differences in the distribution of physicochemical properties of amino acid residues surrounding methylation and non-methylation sites based on the paired Welch's t-test. As shown in Figure 1, from −7 to +7 positions, the ratios of hydrophobic amino acids around methylarginine were 2.3% to 29.2% higher than those of non-methylarginine with *P*-value ≤3.59e-02 (see Table S6). Especially for the +1 position, hydrophobic residues around methylarginine account for 77.3%, about 29.2% higher than those of non-methylarginine (*P*  = 3.84e-09). From −7 to +7 positions, polar and negatively charged residues surrounding non-methylarginine were 1.15% to 7.12% higher than those of methylarginine (*P*<0.05). This analysis reveals that methylarginine and non-methylarginine have distinct physiochemical properties. In fact, some studies suggested that the arginine residue becomes more hydrophobic due to addition of methyl groups and may engage in more van der Waal interactions [8].

**Table 1:** The performance of models trained with various features for methylarginine.

Training features	Sn (%)	Sp (%)	Acc (%)	MCC (%)
SPC	68.96±1.52	92.71±1.09	86.78±0.56	63.78±1.66
PWAA	60.85±0.57	95.51±0.50	86.85±0.41	62.76±1.16
ASA	51.94±0.15	99.81±0.15	87.84±0.12	66.37±0.45
VDWV	56.34±1.22	98.91±0.34	88.24±0.54	67.08±1.81
SPC+PWAA	71.66±2.14	91.87±0.60	86.82±0.75	64.40±2.55
SPC+ASA	65.69±1.22	95.46±0.69	88.01±0.68	66.42±1.93
PWAA+ASA	66.42±2.37	92.13±0.90	85.70±1.02	60.72±2.78
SPC+PWAA+ASA	74.09±1.44	93.35±1.45	88.54±0.64	68.93±1.88
SPC+PWAA+VDWV	74.54±3.56	94.31±1.17	89.37±0.83	71.69±3.79
SPC+PWAA+ASA+VDWV	80.73±1.58	92.28±1.24	89.39±1.35	72.45±1.73

The corresponding measurement was represented as the average value±standard deviation. The window size was 15 and the ratio between positive and negative samples was 1∶3.

**Table 2:** The performance of models trained with various features for methyllysine.

Training features	Sn (%)	Sp (%)	Acc (%)	MCC (%)
SPC	61.69±1.00	99.79±0.16	90.31±0.21	73.44±0.60
PWAA	58.88±0.65	99.46±0.20	89.36±0.27	70.53±0.86
ASA	53.31±3.04	98.34±0.33	87.14±0.82	63.40±2.62
VDWV	54.38±2.49	99.48±0.35	88.26±0.42	67.27±1.16
SPC+PWAA	65.88±1.77	99.79±0.16	91.35±0.43	76.40±1.23
SPC+ASA	64.44±3.32	99.01±0.44	90.40±0.76	73.41±2.23
PWAA+ASA	63.00±3.29	99.34±0.35	90.30±0.76	73.20±2.21
SPC+PWAA+ASA	69.94±2.78	99.11±0.40	91.85±0.64	77.59±1.84
SPC+PWAA+VDWV	68.88±3.74	99.83±0.15	92.13±1.19	78.59±2.54
SPC+PWAA+ASA+VDWV	73.56±2.08	99.11±0.39	92.75±0.25	80.15±0.64

The corresponding measurement was represented as the average value±standard deviation. The window size was 15 and the ratio between positive and negative samples was 1∶3.

While compared with non-methyllysine, the ratios of four different attributive residues around methyllysine have not changed much, as shown in Figure 2, which indicates that the incorporation of methyl groups to the lysine side chain changes the physicochemical properties of the affected residues only slightly. It is worth noting that the ratios of polar residues surrounding methyllysine were 2.81% to 6.03% higher than those of non-methyllysine from −5 to −2 positions (*P*≤8.46e-04). Most enzymes bind the methyllysine in a polar environment, which resembles the ‘carbonyl cage’ of SET domains rather than the hydrophobic pockets of chromo domain-related motifs [40]. The methyl groups are coordinated by a set of electrostatic interactions between polar residues of the protein and the trimethylammonium. CH…O-H bonds form between oxygen on the enzyme's sidechains and methyl groups of the methyllysine [41]. These interactions cumulatively position one of the methyl groups in the vicinity of the iron for hydroxylation to occur [24]. All these researches strengthen the role of surrounding sites in the enzymes' reorganization.

#### VDWV feature analysis

Figure 3 gives the mean values of normalized van der Waals volume (VDWV) of residues around methylation sites and non-methylation sites based on training data**.** From −7 to +7 positions, the mean values of VDWV of residues surrounding methylarginine were lower than those of non-methylarginine, especially for the -1 and +1 position. Most of *P*-values were less than 0.05 (see Table S7), indicating that there was significant difference between the VDWV surrounding methylarginine and that surrounding non-methylarginine. From −7 to −1 positions, there was obvious difference between the VDWV surrounding methyllysine and that surrounding non-methyllysine (*P*≤1.24e-05). This reveals that the upstream residues may have a significant influence on methyllysine.

**Table 3:** Independent test results of PMeS.

Residue type	Number of positivetest data	Number of negativetest data	Sn (%)	Sp (%)	Acc (%)	MCC (%)
Arginine	27	27	85.19±3.64	96.30±3.25	90.74±2.18	81.99±3.95
Lysine	46	46	76.09±1.90	95.65±3.03	85.87±2.49	73.15±3.41

The corresponding measurement was represented as the average value±standard deviation.

**Table 4:** Comparison of PMeS with MASA on the dataset adopted in MASA method.

Prediction methods	Residue type	Training features	Sn (%)	Sp (%)	Acc (%)	MCC (%)
MASA	Arginine	AA+ASA	82.1	87.4	84.8	69.6(a)
	Lysine	AA+ASA	75.1	74.0	74.6	49.2^(b)^
PMeS^(c)^	Arginine	SPC+PWAA+ASA+VDWV	86.18±2.43	90.24±2.33	88.21±1.29	76.61±4.02
	Lysine	SPC+PWAA+ASA+VDWV	83.09±3.14	99.23±0.84	91.16±1.69	83.44±3.07

(a) The MCC for methylarginine in MASA [23] was 79.6%, which was the author’s mistake in calculation. We corrected it for 69.6% by the calculating formula of MCC. ^(b)^The MCC for methyllysine in MASA was 56.1%, which was the author’s mistake in calculation. We corrected it for 49.2% by the calculating formula of MCC. ^(c)^ The corresponding measurement was represented as the average value±standard deviation. Abbreviation: AA, amino acid.

**Table 5:** Comparison of PMeS with Hu’s method on the dataset adopted in Hu's method.

Prediction methods	Residue type	Training features	Sn (%)	Sp (%)	Acc (%)	MCC (%)
Hu's method	Arginine	AAF+PSSM+SD	74.39±2.21	74.11±3.27	74.25±1.46	48.52±2.85
	Lysine	AAF+PSSM+SD	79.73±1.66	74.54±3.61	77.02±1.95	54.28±3.74
PMeS	Arginine	SPC+PWAA+ASA+VDWV	82.03±2.53	84.41±3.82	83.22±3.06	66.57±4.53
	Lysine	SPC+PWAA+ASA+VDWV	79.11±2.98	88.44±2.52	83.78±1.48	68.54±4.79

The corresponding measurement was represented as the average value±standard deviation. Abbreviation: AAF, amino acid factors; PSSM, position specific scoring matrix; SD, structural disorder.

#### PWAA feature analysis

PWAA feature reflects the position information of residues surrounding methylation sites and non-methylation sites. In order to analyze position specific properties, we adopted WebLogo [42] to generate the graphical sequence logo for the relative frequency of the corresponding amino acid at each position around methylation and non-methylation sites. As we can see from Figure 4, the methylated arginines (R) are enriched in arginine-glycine (R–G) regions which are much different from non-methylated arginines. Indeed, motif analysis reveals many arginine methylation are associated with RGG/RXG/RGX [43] or GXXR [20] motifs. The conserved residues at specific sequence sites are under strong selective pressure and therefore are always functional relevant. The type I PRMTs is known to methylate a number of proteins that contain an RGG-motif [44]. The repeated RGG-motif is known as a RNA-binding motif [45], and this also supports the role of arginine methylation in the regulation of mRNA binding [46]. In contrast, no amino acids surrounding methylated lysines (K) are obviously conserved in the current available data (Fig. 4). Therefore, sequence profiles of the flanking regions of methylarginine are more conservative with higher specificity than those of methyllysine.

#### ASA feature analysis

Figure 5 summarizes the average accessible surface area (ASA) formed from the 15-mer methylation sites and the 15-mer non-methylation sites in the constructed data set. Most of the methylation or non-methylation sites (0 position) were located in the highly ASA, which was consistent with those data reported in the literature [35]. The average ASA of neighborhood residues were 23.09% to 39.01% and 25.54% to 49.90% for methylarginine and methyllysine, respectively. The fluctuant range of ASA of residues surrounding methylation sites was bigger than that of non-methylation sites. This implies that the methylation processing might have occurred where the structural surroundings are relatively large variation range. The mean ASA that surrounds the methylarginine exceeded that around non-methylarginine, especially in the −6, −3, 0, +1, +4, +5 and +6 positions (*P*≤5.21e-03, see Table S8). Interestingly, the mean ASA around the methyllysine was slightly below that around non-methyllysine, especially in the −6, −3, −1, +2 and +3 positions (*P*≤3.06e-02). Generally speaking, the ASA of residues around the methylation sites and non-methylation sites have a little difference.

There were two possible reasons for limiting the ASA analysis in the methylation: first, the negative sites were obtained as not being previously experimentally identified; second, the ASA values were predicted by RVP-Net server. Table S9 gives the predicted ASA and experimental ASA of methylation sites with known tertiary structure of protein data bank. There are some differences between the predicted ASA and experimental ASA of methylation sites. The experimental ASA of most methyllysine are more than 30%. In the RVP-Net, the residue is exposed when its ASA is more than 16%. Thus, it seems that it may be important that the methyllysine need be solvent exposed. While the experimental ASA of several methylarginine (eg. P53674, R230 and R231) are lower than 12%. If the experimental ASA of most methylation sites were obtained, we could get a more reliable conclusion.

#### Optimal feature set

When the window size was 15 and the ratio between positive and negative samples was 1∶3, the predictive performance of models trained with various features for methylarginine and methyllysine are shown in Tables 1 and 2, respectively. According to statistical comparison of sensitivity (see Table S10), the model trained with SPC outperformed that trained with PWAA, VDWV or ASA (*P*≤2.21e-03), which was in agreement with the results of above feature analysis. But in general, the models trained with individual features could not effectively distinguish methylation sites from non-methylation sites. However, the predictive performance of the methyllysine model trained with the combination of SPC and PWAA (SPC+PWAA) or SPC, PWAA and ASA (SPC+PWAA+ASA) had some improvements (*P*≤2.48e-02). The predictive performance of the methylarginine model trained with the combination of SPC, PWAA and ASA (SPC+PWAA+ASA) also had some improvements (*P*≤6.92e-04). Furthermore, both methylarginine and methyllysine, the performance of the model trained with SPC+PWAA+ASA+VDWV had been remarkably enhanced (*P*<0.05). This demonstrated that all four types of features contributed to distinguishing between methylation sites and non-methylation sites. There was a strong complementary effect among these features. Henceforth, the combination of SPC, PWAA, ASA and VDWV was selected as an optimal feature set to learn the predictive model.

Moreover, we noticed that the performance of the predictive models on arginine was much better than on lysine in Table 1 and 2. This observation agrees with the above feature analysis, which the difference of the physiochemical properties between methylarginine and non-methylarginine is more obvious than that of methyllysine and non-methyllysine, and the sequence pattern of methylarginine is more conservative with higher specificity than that of methyllysine.

### Investigation of Window Sizes

For each methylation or non-methylation sites, its profile feature and ASA feature were taken from a sequence fragment containing the *n* nearest residues (spatially); thus, it is crucial to confirm the appropriate window size and to realize its effects on the prediction performance. The predictive performance of models trained with different window sizes (9 to 19) are illustrated in Tables S11 and S12, where training feature was SPC+PWAA+ASA+VDWV and the ratio between positive and negative samples was 1∶3. The results showed that the window size had much more impact on the *Sn* and *MCC* than on the *Sp* and *Acc*, especially for methylarginine. Based on statistical comparison of sensitivity (see Table S13), there were significant differences between the methylarginine model with window size of 15 and those of 9, 13, 17, 19 (*P*≤3.29e-03). The methyllysine model with window size of 15 outperformed that with window sizes of 9, 11, and 19 (*P*≤1.25e-02). There was no statistical difference among the methyllysine model with window sizes of 13, 15 and 17 (*P*>1.44e-01). Based on the computational efficiency and overall performance of the models trained with different window length, 15-mer was adopted as the feasible window size for the two methylation residues in this study.

### Investigation of the Ratios between Positive Samples and Negative Samples

As we can see from the Table 1 and 2, the *Sp* and *Acc* were relatively stable on different features, whereas the *Sn* and *MCC* fluctuated wildly, and it was relatively hard to get a higher sensitivity when the ratio of positive samples to negative samples was 1∶3. This is because the positive examples are extremely few and one incorrect prediction leads to a large decrease on sensitivity, and a larger negative set would cause the trained model preferentially to predict negative data correctly, driven by the requirement to maximize accuracy. Thus, it is very important to use a suitable ratio between positive samples and negative samples to construct the prediction model. As shown in Tables S14 and S15, after the ratio between positive and negative samples arrived at 1∶5, the *MCC* of the predictive models using different ratios of positive and negative samples decreased with increasing the size of the negative set (*P*≤2.38e-02, see Table S16). The best performance of methylarginine models was obtained when the ratio between positive and negative samples was 1∶1 (*P*≤1.42e-02). The corresponding *Sn*, *Sp*, *Acc* and *MCC* were 92.45%, 93.18%, 92.82% and 85.69%, respectively. For methyllysine, when the ratio between positive and negative samples were 1∶1 and 1∶3, there was no statistical difference based on *MCC* comparison (*P* = 5.43e-02). Except for 1∶3, when the ratio between positive and negative samples was 1∶1 the best performance of methyllysine models was obtained (*P*≤1.85e-02), the *Sn*, *Sp*, *Acc* and *MCC* were 84.38%, 93.94%, 89.16% and 78.68%, respectively. Given the narrowing of the gap between the sensitivity and the specificity, 1∶1 was as the suitable ratio between positive samples and negative samples to construct the optimal predictive model PMeS.

### Investigation of the Robustness of PMeS

To test the robustness of our predictive model PMeS, the self-consistency validation, leave-one-out validation and K-fold cross-validation were calculated. Table S17 presents the three test performances of methylarginine model. Based on *MCC* comparison (see Table S18), there was no statistical difference among different cross-validation (*P*≥6.29e-01). Importantly, it is proposed that the leave-one-out test might overfit in small samples, whereas the K-fold cross-validation should do better [47]. However, we observed that the leave-one-out test results were quite similar with 4-, 6-, 8-, 10-fold cross-validations, which demonstrated the robustness and stability of the PMeS. One vital factor that could result in misleadingly high prediction performance and possibly influence prediction stability is sequence homology in training dataset [48]. As described in the Data collection section, we carried out homology reducing process on training dataset. This data preprocessing might be helpful to enhance the robustness of the PMeS.

### Independent Test

Moreover, to validate our algorithm against other sources of methylation data from experimental papers, we collected 46 experimental methyllysine sites and 27 experimental methylarginine sites from scientific literatures to construct the independent test sets (see Tables S19 and S20). None of independent test proteins was included in the training dataset. As shown in Table 3, besides the *Sn*, the other three measurements of independent test for methylarginine were quite similar with those of training test (*P*≥1.12e-01, see Table S21). For methyllysine, the *Sp* of the independent test was slightly higher than that of training test (*P*  = 2.40e-03), the other three measurements of independent test were 3.29% to 8.29% lower than those of training test (*P*≤5.50e-03). If the performance of the independent test is much worse than that of training test, then the trained model may be over-fitting for the training data. Generally, the performance of the independent test was just a little lower than those of training test, which was also acceptable. Moreover, the negative sites were obtained as not being previously experimentally identified, which might be a possible reason for influencing the predictive results.

### Comparisons with Existing Methods

In order to further evaluate the prediction performance of the PMeS method objectively, we made comparisons with other methylation predictor. Here the performance of the PMeS on the dataset adopted in MASA [23] and Hu's method [24] were evaluated as shown in Tables 4 and 5, respectively. For methyllysine, the four measurements in PMeS were 7.99% to 34.24% higher than those in MASA (*P*≤4.18e-04, see Table S22). For methylarginine, besides the *Sp* (*P*  = 2.21e-01), the other three measurements in PMeS were 3.41% to 7.01% higher than those in MASA (*P*≤3.52e-02). Compared with the training features (AA+ASA) in MASA, our significant improvements can be attributed to the adoption of the physicochemical properties of residues, as elucidated in the above feature analysis, the physicochemical properties are effective in identifying methylation status. Similarly, for methyllysine, except the *Sn* (*P*  = 4.70e-01, see Table S23), the other three measurements in PMeS were 6.76% to 14.26% higher than those in Hu's method (*P*<4.67e-02). For methylarginine, the four measurements in PMeS were so much better than those in Hu's method (*P*<0.05). Compared with Hu's method, our improvements may come from SPC feature. In some problems (e.g. HIV protease), where the training set could be not completely representative of the test set, the sparse orthonormal representation works very well [49]. In summary, the PMeS outperformed MASA and Hu's method, which justified the effectiveness of SPC+PWAA+ASA+VDWV as feature for methylation sites prediction.

### Conclusion

Methylation prediction methods in previous studies, such as MeMo [21], BPB-PPMS [22] and MASA [23], have focused only on orthogonal encoding scheme to represent protein sequence information, where do not contain preferences on amino acids or position information and physicochemical properties of residues. However, the enhanced feature encoding scheme PMeS in this study incorporated the amino acid sequence, position information, physicochemical properties of residues with structural characteristic to improve the prediction of protein methylation sites. Feature analysis showed that methylation and non-methylation sites had distinct physiochemical properties, and the SPC, VDWV, PWAA and ASA features all contributed to the methylation prediction. The cross-validation results demonstrated that PMeS achieved a promising performance and outperformed other methylation prediction tools. In addition, the PMeS had a greater robustness. It can be anticipated that the PMeS might be useful to guide future experiments needed to identify potential methylation sites in proteins of interest. Datasets and Matlab code can be downloaded from our website (http://bioinfo.ncu.edu.cn/inquiries_PMeS.aspx).

## Supporting Information

Table S1 **246 experimentally identified methylarginine sites in 98 proteins were extracted from UniProtKB/Swiss-Prot database.**
(DOC)

Table S2 **367 experimentally identified methyllysine sites in 137 proteins were extracted from UniProtKB/Swiss-Prot database.**
(DOC)

Table S3 **155 methylarginine sites in 68 proteins were extracted from PhosphoSitePlus.**
(DOC)

Table S4 **147 methyllysine sites in 78 proteins were extracted from PhosphoSitePlus.**
(DOC)

Table S5 **The normalized van der Waals volume of 20 kinds of amino acids.**
(DOC)

Table S6 **The distribution of physicochemical properties of residues around methylation sites and non-methylation sites was compared via** ***P*** **-values on the paired Welch's t-test.**
(DOC)

Table S7 **Average van der Waals volume (VDWV) of residues around methylation sites and non-methylation sites was compared via** ***P*** **-values on the paired Welch's t-test.**
(DOC)

Table S8 **Average accessible surface area (ASA) of residues around methylation sites and non-methylation sites was compared via** ***P*** **-values on the paired Welch's t-test.**
(DOC)

Table S9 **The list of proteins containing experimental methylation sites which are located in the protein regions with known tertiary structure of protein data bank (PDB).**
(DOC)

Table S10 **The predictive performance of model trained with different features was compared via** ***P*** **-values on the paired Welch's t-test.**
(DOC)

Table S11 **The performance of models trained with different window sizes for methylarginine.**
(DOC)

Table S12 **The performance of models trained with different window sizes for methyllysine.**
(DOC)

Table S13 **The predictive result of models with different window sizes was compared via** ***P*** **-values on the paired Welch's t-test.**
(DOC)

Table S14 **The performance of models trained with different ratio of positive to negative samples for methylarginine.**
(DOC)

Table S15 **The performance of models trained with different ratio of positive to negative samples for methyllysine.**
(DOC)

Table S16 **The predictive result of models with different ratios of positive to negative samples was compared via** ***P*** **-values on the paired Welch's t-test.**
(DOC)

Table S17 **The performance of the methylarginine model based on self-consistency, K-fold (4-, 6-, 8- and 10-fold) cross-validation and leave-one-out validation.**
(DOC)

Table S18 **MCC of methylarginine model by different cross-validation was compared via** ***P*** **-values on the paired Welch's t-test.**
(DOC)

Table S19 **We collected 46 experimentally identified methyllysine sites in 39 unique proteins from the scientific literature (PubMed).**
(DOC)

Table S20 **We collected 27 experimentally identified methylarginine sites in 24 unique proteins from the scientific literature (PubMed).**
(DOC)

Table S21 **Statistical comparison of training test with independent test based on the paired Welch's t-test.**
(DOC)

Table S22 **Statistical comparison of PMeS with MASA on the dataset adopted in MASA method.**
(DOC)

Table S23 **Statistical comparison of PMeS with Hu's method on the dataset adopted in Hu's method.**
(DOC)
