# Supplementary material for: PMeS: Prediction of Methylation Sites Based on Enhanced Feature Encoding Scheme
Source: PLoS One. 2012 Jun 15;7(6):e38772. doi: 10.1371/journal.pone.0038772 (PMC3376144; doi:10.1371/journal.pone.0038772)
Supplement: Table S1 — 246 experimentally identified methylarginine sites in 98 proteins were extracted from UniProtKB/Swiss-Prot database. (DOC) [file pone.0038772.s001.doc]

**Table S1. 246 experimentally identified methylarginine sites in 98 proteins were extracted from UniProtKB/Swiss-Prot database.**

| Accession | Protein name_species | Residue position |
| --- | --- |
| Q9NR22 | ANM8_HUMAN | 58,73 |
| Q9DCB4 | ARP21_MOUSE | 650 |
| Q86WR7 | CJ047_HUMAN | 252,320 |
| P05813 | CRBA1_HUMAN | 137 |
| P02489 | CRYAA_HUMAN | 21 |
| P17844 | DDX5_HUMAN | 502 |
| Q15717 | ELAV1_HUMAN | 217 |
| P26378 | ELAV4_HUMAN | 243 |
| Q28165 | PABP2_BOVIN | 238,259,263,265,267,269,277,279,287,289,291,294,296,298 |
| Q9NZB2 | F120A_HUMAN | 982 |
| P22509 | FBRL_RAT | 8,15,21,24,28,31 |
| Q54G21 | FKBP5_DICDI | 314 |
| Q06787 | FMR1_HUMAN | 544 |
| P51114 | FXR1_HUMAN | 445 |
| Q9UN86 | G3BP2_HUMAN | 457,468 |
| Q3ZU82 | GOGA5_RAT | 27,89 |
| P62805 | H4_HUMAN | 4 |
| P68431 | H31_HUMAN | 3,9,18 |
| Q71DI3 | H32_HUMAN | 3,9,18 |
| Q6PI79 | H33_XENLA | 3,18 |
| O14979 | HNRDL_HUMAN | 408 |
| P31942 | HNRH3_HUMAN | 121,287 |
| P38159 | HNRPG_HUMAN | 185 |
| Q00839 | HNRPU_HUMAN | 50,733,739 |
| Q12906 | ILF3_HUMAN | 609 |
| P08199 | NUCL_MESAU | 656,660,666,670,674,680,688,692,695 |
| Q36736 | KM11_LEIDO | 45 |
| P02687 | MBP_BOVIN | 106 |
| P83487 | MBP_HORSE | 108 |
| P06906 | MBP_PANTR | 107 |
| P25274 | MBP_RABIT | 105 |
| P07962 | MCRA_METBF | 285 |
| P08729 | K2C7_HUMAN | 20 |
| Q13310 | PABP4_HUMAN | 518 |
| Q9JMB7 | PIWL1_MOUSE | 14,49,371 |
| P98179 | RBM3_HUMAN | 105 |
| P0CX53 | RL12_YEAST | 67 |
| P09867 | ROA1_BOVIN | 194,206,225 |
| P04256 | ROA1_RAT | 194,206,225 |
| P51991 | ROA3_HUMAN | 52,246 |
| P80350 | ROAB_ARTSA | 119,122,192 |
| P23246 | SFPQ_HUMAN | 7,9,19,25,571,681,693 |
| Q07955 | SFRS1_HUMAN | 93,97,109 |
| P62318 | SMD3_HUMAN | 97 |
| P69697| TAT_HV1B1 | 52,53 |
| P07437 | TBB5_HUMAN | 318 |
| Q86V81 | THOC4_HUMAN | 50,204,220 |
| Q6AY25 | TMED3_RAT | 103 |
| P62995 | TRA2B_HUMAN | 241 |
| Q8WWM7 | ATX2L_HUMAN | 361 |
| Q7Q2B7 | CARM1_ANOGA | 482 |
| P05179 | CP2C7_RAT | 144 |
| P53674 | CRBB1_HUMAN | 230,231 |
| P02511 | CRYAB_HUMAN | 22,50 |
| P06746 | DPOLB_HUMAN | 83,152 |
| P70372 | ELAV1_MOUSE | 217 |
| O09032 | ELAV4_RAT | 236 |
| Q01844 | EWS_HUMAN | 300,302,304,309,314,317,321,455,464,471,490,494,500,503,506,563,565,572,575,581,589,592,596,600,603,607,615,633,636 |
| P22508 | FBRL_PHYPO | 5,11,16,19 |
| Q60487 | FGF2_CAVPO | 4,6,8 |
| Q8K4C0 | FMO5_RAT | 5 |
| P35637 | FUS_HUMAN | 216,218 |
| Q13283 | G3BP1_HUMAN | 435,447,460,465 |
| P03347| GAG_HV1B1 | 387,409 |
| Q9R064 | GORS2_RAT | 30,47 |
| P62806 | H4_MOUSE | 4 |
| P68433 | H31_MOUSE | 3,9,18 |
| P84233 | H32_XENLA | 3,18 |
| P17096 | HMGA1_HUMAN | 26,58,60 |
| P31943 | HNRH1_HUMAN | 217,233 |
| Q14103 | HNRPD_HUMAN | 345 |
| P61978 | HNRPK_HUMAN | 296,299 |
| P07687 | HYEP_RAT | 295 |
| P04264 | K2C1_HUMAN | 82 |
| Q07666 | KHDR1_HUMAN | 45,52,291,304,310,315, 320,325, 331,340,346 |
| P29996| LHDAG_HDVD3 | 13 |
| P15720 | MBP_CHICK | 106 |
| P02686 | MBP_HUMAN | 241 |
| P81558 | MBP_PIG | 107 |
| P02688 | MBP_RAT | 131 |
| P11558 | MCRA_METTM | 271 |
| P11940 | PABP1_HUMAN | 455,460,493 |
| Q09472 | EP300_HUMAN | 580,604,2142 |
| Q8CDG1 | PIWL2_MOUSE | 74,95,100,163,549 |
| Q92804 | RBP56_HUMAN | 206,483,570 |
| Q13151 | ROA0_HUMAN | 291 |
| P09651 | ROA1_HUMAN | 194,206,225 |
| P22626 | ROA2_HUMAN | 203,213 |
| Q99729 | ROAA_HUMAN | 245,322 |
| P14678 | RSMB_HUMAN | 108,112,147 |
| Q07666 | SAM68_HUMAN | 45,52,291,304,310,315,320,325,331,340,346 |
| P0C6L3| SHDAG_HDVD3 | 13 |
| O00267 | SPT5H_HUMAN | 681,696,698 |
| P68363 | TBA1B_HUMAN | 339 |
| P19814 | TGON3_RAT | 74 |
| O08583 | THOC4_MOUSE | 50,203,218 |
| Q63584 | TMEDA_RAT | 171,176 |
| P12346 | TRFE_RAT | 42 |
