# Supplementary material for: PMeS: Prediction of Methylation Sites Based on Enhanced Feature Encoding Scheme
Source: PLoS One. 2012 Jun 15;7(6):e38772. doi: 10.1371/journal.pone.0038772 (PMC3376144; doi:10.1371/journal.pone.0038772)
Supplement: Table S2 — 367 experimentally identified methyllysine sites in 137 proteins were extracted from UniProtKB/Swiss-Prot database. (DOC) [file pone.0038772.s002.doc]

**Table S2. 367 experimentally identified methyllysine sites in 137 proteins were extracted from UniProtKB/Swiss-Prot database.**

| Accession | Protein name_species | Residue position |
| --- | --- |
| P14909 | AAT_SULSO | 203,385 |
| P39462 | ADH_SULSO | 11,213 |
| P22498 | BGAL_SULSO | 116,135,273,311,332 |
| P11118 | CALM_EUGGR | 116,149 |
| Q95NR9 | CALM_METSE | 116 |
| P04573 | CAVP_BRALA | 96,117 |
| P53674 | CRBB1_HUMAN | 235 |
| P26998 | CRBB3_HUMAN | 128 |
| P0C835 | CREN7_SULSH | 16 |
| P00044 | CYC1_YEAST | 78 |
| P00059 | CYC_ABUTH | 80,94 |
| P00053 | CYC_CANSA | 80,94 |
| P00077 | CYC_CRION | 4,83 |
| P00076 | CYC_EUGGR | 85 |
| P00072 | CYC_FAGES | 80,94 |
| P00070 | CYC_HELAN | 81,95 |
| Q0DI31 | CYC_ORYSJ | 81,95 |
| P00047 | CYC_THELA | 80,94 |
| P80053 | DHE2_SULSO | 254,260,372,391,392 |
| P61991 | DN71_SULSO | 5,7 |
| P39476 | DN72_SULSO | 5,7,61,63,64 |
| P68105 | EF1A1_RABIT | 36,55,79,165,318 |
| Q5VTE0 | EF1A3_HUMAN | 36,55,79,165,318 |
| P02994 | EF1A_YEAST | 30,79,316,390 |
| P0CE48 | EFTU2_ECOLI | 57 |
| P55907 | FER1_SULTO | 30 |
| P81541 | FER_ACIIN | 29 |
| P00219 | FER_SULAC | 29 |
| P81539 | FERB_SULME | 29 |
| O75367 | H2AY_HUMAN | 18,123 |
| P58876 | H2B1D_HUMAN | 47,58,109 |
| Q99877 | H2B1N_HUMAN | 47,58,109 |
| Q9FFC0 | H2B10_ARATH | 12 |
| P08898 | H3_CAEEL | 5,10,28,37,80 |
| P80322 | TNNC_BRALA | 30 |
| P62803 | H4_BOVIN | 21 |
| P62805 | H4_HUMAN | 21 |
| P82888 | H4_OLILU | 80 |
| P62804 | H4_RAT | 21 |
| P10412 | H14_HUMAN | 26 |
| Q42681 | H31_CHLRE | 5,10,28,36,37 |
| P59226 | H32_ARATH | 5,10,19,24,28,37 |
| P84229 | H32_CHICK | 5,10,15,28,37,80 |
| Q6LCW8 | H32_CHLRE | 5,10,28,36,37 |
| P69246 | H32_MAIZE | 5,10,19,24,28 |
| P68429 | H32_MEDSA | 5,10,15,19,28 |
| P84233 | H32_XENLA | 5,10,19,28,37 |
| P84249 | H33_DROME | 5,10,15,28,37,38,80 |
| Q6PI79 | H33_XENLA | 5,10,19,24,28,37 |
| Q41811 | H43_MAIZE | 21 |
| O14979 | HNRDL_HUMAN | 161 |
| P04264 | K2C1_HUMAN | 276 |
| P10587 | MYH11_CHICK | 128 |
| P13538 | MYSS_CHICK | 36,131,552 |
| P04637 | P53_HUMAN | 370,372 |
| P68696 | PRO1A_ACACA | 104 |
| P19984 | PROF2_ACACA | 104 |
| P27064 | RBL_CUCSA | 14 |
| P04992 | RBL_PETHY | 14 |
| P25079 | RBL_SOLTU | 14 |
| P0A7K2 | RL7_ECOLI | 82 |
| Q86L05 | RL10A_DICDI | 122 |
| P0CX53 | RL12_YEAST | 4,11 |
| P25886 | RL29_RAT | 5 |
| P62986 | RL40_RAT | 98 |
| P22626 | ROA2_HUMAN | 104 |
| Q12962 | TAF10_HUMAN | 189 |
| Q7M1B9 | THIO_CHLAA | 105 |
| P05547 | TNNI_PONLE | 142,146 |
| P18281 | ACTO_ACACA | 35,72 |
| P05141 | ADT2_HUMAN | 52 |
| P62157 | CALM_BOVIN | 116 |
| P62158 | CALM_HUMAN | 116 |
| P07463 | CALM_PARTE | 14,116 |
| P00889 | CISY_PIG | 395 |
| P43320 | CRBB2_HUMAN | 42,68,121 |
| P22914 | CRBS_HUMAN | 7 |
| P22914 | CRYAA_HUMAN | 88 |
| P00107 | CYC6_PAVLU | 24 |
| P00063 | CYC_ACENE | 80,94 |
| P00078 | CYC_CRIFA | 4,83 |
| P00043 | CYC_DEBHA | 79 |
| P22342 | CYC_EUGVI | 85 |
| P00042 | CYC_HANAN | 61,78,79 |
| P00048 | CYC_NEUCR | 77 |
| P00046 | CYC_SCHPO | 77 |
| P00068 | CYC_WHEAT | 80,94 |
| P13123 | DN71_SULAC | 5,7 |
| P13125 | DN72_SULAC | 7,63,64 |
| P68104 | EF1A1_HUMAN | 36,55,79,165,318 |
| Q71V39 | EF1A2_RABIT | 55,165 |
| P02993 | EF1A_ARTSA | 36,55,79,219,318 |
| P0CE47 | EFTU1_ECOLI | 57 |
| P53991 | FENR_CHLRE | 118,124,170 |
| P49949 | FER_ACIAM | 29,101 |
| P81542 | FER_METPR | 30 |
| P81543 | FERA_SULME | 29 |
| P04406 | G3P_HUMAN | 5,66,194,215,227,260,263,334 |
| P62807 | H2B1C_HUMAN | 47,58,109 |
| O60814 | H2B1K_HUMAN | 47,58,109 |
| Q9LZT0 | H2B7_ARATH | 2,4 |
| P40283 | H2B11_ARATH | 2,4 |
| P02299 | H3_DROME | 5,10,15,28,37,38,80 |
| P61830 | H3_YEAST | 5,10,15,19,24,28,37,80 |
| P62784 | H4_CAEEL | 21 |
| Q4R362 | H4_MACFA | 21 |
| P83865 | H4_PENVA | 21,32 |
| P16403 | H12_HUMAN | 34 |
| P68432 | H31_BOVIN | 5,10,19,24,28,37,38,65,80,123 |
| P68431 | H31_HUMAN | 5,10,19,24,28,37,38,57,65,80,123 |
| P68433 | H31_MOUSE | 5,10,19,24,28,37,38,65,80,123 |
| Q16695 | H31T_HUMAN | 5,10,19,28,37,65,80,123 |
| P08903 | H32_ENCAL | 5,10,19,24,28 |
| Q71DI3 | H32_HUMAN | 5,10,19,24,28,37,57,65,80,123 |
| P68427 | H32_PEA | 5,10,15,19,24,28 |
| P59169 | H33_ARATH | 5,10,19,24,28,37 |
| P10651 |H33_SCHPO | 5,10,15,19,24,28,37,80 |
| O15819 | H33A_DICDI | 5,10,19,24,28,40,83 |
| P43320 | CRBB2_HUMAN | 42,68,121 |
| Q14103 | HNRPD_HUMAN | 119 |
| P16862 | K6PF2_YEAST | 180 |
| O00159 | MYO1C_HUMAN | 383 |
| P11940 | PABP1_HUMAN | 299 |
| Q13310 | PABP4_HUMAN | 361 |
| Q95VF7 | PRO1B_ACACA | 104 |
| P11416 | RARA_MOUSE | 347 |
| P04717 | RBL_PEA | 14 |
| P27065 | RBL_SOLLC | 14 |
| P02393 | RL7_DESVM | 77,88 |
| P07472 | RL7_HALEU | 84 |
| P0A7J7 | RL11_ECOLI | 4,40 |
| P47914 | RL29_HUMAN | 5 |
| Q969Q0 | RL36L_HUMAN | 53 |
| P62986 | RL44_YEAST | 40,55 |
| P23246 | SFPQ_HUMAN | 314 |
| Q9SE35| SIL1_CYLFU | 144,155,166,185,204,223,242 |
| P07041 | H3_NEUCR | 5,10,15,19,24,28,37,80 |
