# Supplementary material for: PMeS: Prediction of Methylation Sites Based on Enhanced Feature Encoding Scheme
Source: PLoS One. 2012 Jun 15;7(6):e38772. doi: 10.1371/journal.pone.0038772 (PMC3376144; doi:10.1371/journal.pone.0038772)
Supplement: Table S3 — 155 methylarginine sites in 68 proteins were extracted from PhosphoSitePlus. (DOC) [file pone.0038772.s003.doc]

**Table S3. 155 methylarginine sites in 68 proteins were extracted from PhosphoSitePlus.**

| Accession | Protein name_species | Residue position |
| --- | --- |
| P50554 | GABT_RAT | 144 |
| Q01196 | RUNX1_HUMAN | 206,210 |
| Q92793 | CBP_HUMAN | 714,742,768 |
| Q12778 | FOXO1_HUMAN | 251,253 |
| P29033 | CXB2_HUMAN | 75 |
| P11598 | PDIA3_RAT | 329,363 |
| P45700 | MA1A1_MOUSE | 103,165 |
| Q15596 | NCOA2_HUMAN | 501 |
| P59240| NPHP4_MOUSE | 919,922 |
| P25444| RS2_MOUSE | 34,36,38,40,42,44,46,48 |
| Q9ET30| TM9S3_MOUSE | 115,205 |
| Q9UQE7 | SMC3_HUMAN | 72,155,236,272,323,644 |
| P52633 | STAT6_MOUSE | 27 |
| P0C7T6 | ATX1L_MOUSE | 493 |
| P11506 | AT2B2_RAT | 718 |
| P11275 | KCC2A_RAT | 220 |
| Q64458 | CP2CT_MOUSE | 144 |
| Q9Y5B0 | CTDP1_HUMAN | 913,916 |
| Q1KMD3 | HNRL2_HUMAN | 656 |
| Q14532 | K1H2_HUMAN | 113 |
| P08644 | RASK_RAT | 41 |
| Q9DD20 | MET7B_MOUSE | 230 |
| P13591 | NCAM1_HUMAN | 130 |
| Q9Y6Q9 | NCOA3_HUMAN | 251 |
| Q561S0 | NDUAA_RAT | 139,326,332 |
| Q9Y3R4 | NEUR2_HUMAN | 314 |
| O70343 | PRGC1_MOUSE | 365 |
| P48552 | NRIP1_HUMAN | 239,649,947 |
| O35182 | SMAD6_MOUSE | 74 |
| Q04887 | SOX9_MOUSE | 74,152,177,178,179 |
| P42224 | STAT1_HUMAN | 31 |
| Q02563 | SV2A_RAT | 48 |
| P33981 | TTK_HUMAN | 467 |
| Q05086 | UBE3A_HUMAN | 294 |
| Q12888 | TP53B_HUMAN | 1162,1396,1398,1400,1401,1403 |
| P45481 | CBP_MOUSE | 623,624 |
| P98082 | DAB2_HUMAN | 64 |
| P50398 | GDIA_RAT | 208 |
| Q62931 | GOSR1_RAT | 112 |
| P62806 | H4_MOUSE | 36 |
| Q09325 | MGAT1_RAT | 31,36 |
| P97546 | NPTN_RAT | 149 |
| P04637 | P53_HUMAN | 110,209,213,333,335,337 |
| P23396 | RS3_HUMAN | 64,65,67 |
| Q14683 | SMC1A_HUMAN | 92,160 |
| Q15554 | TERF2_HUMAN | 13,17,18,21,25,27,28,30 |
| P58021 | TM9S2_MOUSE | 65,90 |
| P05026 | AT1B1_HUMAN | 118 |
| P21272 | CEBPB_RAT | 114 |
| P00403 | COX2_HUMAN | 82 |
| P10634 | CP2DQ_RAT | 333,477 |
| P09038 | FGF2_HUMAN | 82,84,86,96,98,108,110,112 |
| P29477 | NOS2_MOUSE | 716,1041 |
| Q62976 | KCMA1_RAT | 805 |
| Q63356 | MYO1E_RAT | 635 |
| P19338 | NUCL_HUMAN | 656,660,666,670,673 |
| O09000 | NCOA3_MOUSE | 839,844,1178,1184,1195 |
| O09111 | NDUBB_MOUSE | 127 |
| O75306 | NDUS2_HUMAN | 323 |
| Q9JJW6 | REFP2_MOUSE | 24,32,37,40,166,171,173,181,186,188 |
| Q5XI73 | GDIR1_RAT | 111,152,180 |
| Q15020 | SART3_HUMAN | 906 |
| P62314 | SMD1_HUMAN | 98,100,102,104,106,108,110,112 |
| Q8N3U4 | STAG2_HUMAN | 1066 |
| P42227 | STAT3_MOUSE | 31 |
| P19474 | RO52_HUMAN | 324 |
| P68370 | TBA1A_RAT | 64 |
| P31000 | VIME_RAT | 145 |
