# Supplementary material for: PMeS: Prediction of Methylation Sites Based on Enhanced Feature Encoding Scheme
Source: PLoS One. 2012 Jun 15;7(6):e38772. doi: 10.1371/journal.pone.0038772 (PMC3376144; doi:10.1371/journal.pone.0038772)
Supplement: Table S4 — 147 methyllysine sites in 78 proteins were extracted from PhosphoSitePlus. (DOC) [file pone.0038772.s004.doc]

**Table S4. 147 methyllysine sites in 78 proteins were extracted from PhosphoSitePlus.**

| Accession | Protein name_species | Residue position |
| --- | --- |
| P50554 | GABT_RAT | 142 |
| Q96G01 | BICD1_HUMAN | 45 |
| Q13185 | CBX3_HUMAN | 21,44,143 |
| P11442 | CLH_RAT | 245,246 |
| P98082 | DAB2_HUMAN | 163,173 |
| Q8CGP5 | H2A1F_MOUSE | 119 |
| Q6P7R8 | DHB12_RAT | 155,156 |
| Q9NSB4 | KRT82_HUMAN | 137,207,354 |
| P02688 | MBP_RAT | 129 |
| P16290 | PGAM2_RAT | 174 |
| Q2QD09 | Q2QD09_HUMAN | 194 |
| Q9HD15 | SRA1_HUMAN | 218 |
| P33981 | TTK_HUMAN | 385 |
| P68363 | TBA1B_HUMAN | 394 |
| Q12888 | TP53B_HUMAN | 135,1175,1626 |
| P06687 | AT1A3_RAT | 716,717,763,764 |
| Q05826 | CEBPB_CHICK | 39 |
| P08413 | KCC2B_RAT | 221 |
| P15924 | DESP_HUMAN | 1802 |
| Q01094 | E2F1_HUMAN | 185 |
| P03372 | ESR1_HUMAN | 302 |
| P10860 | DHE3_RAT | 346,352 |
| P43276 | H15_MOUSE | 21,22,23 |
| P43274 | H14_MOUSE | 17,21,22,23 |
| Q07133 | H1T_MOUSE | 111 |
| Q8R1M2 | H2AJ_MOUSE | 119 |
| Q6GSS7 | H2A2A_MOUSE | 100 |
| P08238 | HS90B_HUMAN | 347 |
| P08644 | RASK_RAT | 42 |
| P13010 | XRCC5_HUMAN | 702 |
| P42123 | LDHB_RAT | 58,60 |
| Q15596 | NCOA2_HUMAN | 705 |
| P97546 | NPTN_RAT | 147 |
| Q2NKX8 | ERC6L_HUMAN | 1156 |
| P61289 | PSME3_HUMAN | 121,212 |
| Q8CBD1 | NRIP1_MOUSE | 591,653,778 |
| Q14683 | SMC1A_HUMAN | 149,1133 |
| P60174 | TPIS_HUMAN | 194 |
| P17948 | VGFR1_HUMAN | 831 |
| P20648 | ATP4A_HUMAN | 31 |
| Q86X55 | CARM1_HUMAN | 276 |
| P45973 | CBX5_HUMAN | 32,40,91 |
| P13233 | CN37_RAT | 27,29 |
| P16402 | H13_HUMAN | 65,98,107 |
| P07305 | H10_HUMAN | 12,82,102,108,155 |
| P34931 | HS71L_HUMAN | 189 |
| O43790 | KRT86_HUMAN | 193,222,377 |
| Q9H1E3 | NUCKS_HUMAN | 9,64,175,218 |
| Q62879 | Q62879_RAT | 152 |
| Q63644 | ROCK1_RAT | 462 |
| P19474 | RO52_HUMAN | 138 |
| P68370 | TBA1A_RAT | 60 |
| Q922F4 | TBB6_MOUSE | 103 |
| Q13315 | ATM_HUMAN | 1126,1656 |
| P28033 | CEBPB_MOUSE | 39 |
| Q6P6T1 | C1S_RAT | 586 |
| P83916 | CBX1_HUMAN | 9,35 |
| P78527 | PRKDC_HUMAN | 3372 |
| A2ABF8 | A2ABF8_HUMAN | 242 |
| P50398 | GDIA_RAT | 210 |
| Q63226 | GRID2_RAT | 38,39 |
| P16401 | H15_HUMAN | 27,37 |
| P43275 | H11_MOUSE | 17,23 |
| P22752 | H2A1_MOUSE | 119 |
| Q8CGP7 | H2A1K_MOUSE | 119 |
| P17096 | HMGA1_HUMAN | 23,31,46,55,62,65,67,71,74 |
| P29477 | NOS2_MOUSE | 422 |
| P12956 | XRCC6_HUMAN | 92,114,207 |
| P04642 | LDHA_RAT | 42,57,59 |
| P51608 | MECP2_HUMAN | 119,210 |
| Q9Y6Q9 | NCOA3_HUMAN | 840,1091 |
| Q92831 | KAT2B_HUMAN | 78,89,638,671,672,692 |
| Q63598 | PLST_RAT | 447 |
| Q5XI73 | GDIR1_RAT | 50,52 |
| Q9UQE7 | SMC3_HUMAN | 245,409,427,429,445,486,997 |
| Q8WVM7 | STAG1_HUMAN | 333,618 |
| Q5XIF6 | TBA4A_RAT | 40 |
| P31000 | VIME_RAT | 143 |
