# Supplementary material for: PMeS: Prediction of Methylation Sites Based on Enhanced Feature Encoding Scheme
Source: PLoS One. 2012 Jun 15;7(6):e38772. doi: 10.1371/journal.pone.0038772 (PMC3376144; doi:10.1371/journal.pone.0038772)
Supplement: Table S5 — The normalized van der Waals volume of 20 kinds of amino acids. (DOC) [file pone.0038772.s005.doc]

**Table S5. The normalized van der Waals volume of 20 kinds of amino acids.**

| Amino acid | normalized van der Waals volume | Amino acid | normalized van der Waals volume |
| --- | --- | --- | --- |
| A | 1.00 | M | 4.43 |
| C | 2.43 | N | 2.95 |
| D | 2.78 | P | 2.72 |
| E | 3.78 | Q | 3.95 |
| F | 5.89 | R | 6.13 |
| G | 0.00 | S | 1.60 |
| H | 4.66 | T | 2.60 |
| I | 4.00 | V | 3.00 |
| K | 4.77 | W | 8.08 |
| L | 4.00 | Y | 6.47 |
