# Supplementary material for: PMeS: Prediction of Methylation Sites Based on Enhanced Feature Encoding Scheme
Source: PLoS One. 2012 Jun 15;7(6):e38772. doi: 10.1371/journal.pone.0038772 (PMC3376144; doi:10.1371/journal.pone.0038772)
Supplement: Table S6 — The distribution of physicochemical properties of residues around methylation sites and non-methylation sites was compared via P -values on the paired Welch's t-test. (DOC) [file pone.0038772.s006.doc]

**Table S6. The distribution of physicochemical properties of residues around methylation sites and non-methylation sites was compared via *P*-values on the paired** **Welch's t-test. For the entry at row *i*, column *j* of the table, there is statistical difference when *P*$\leq$0.05, or else there isn’t significantly different. G1 is** **hydrophobic residue, G2 is** **polar residue, G3 is positively charged residue, and G4 is negatively charged residue.**

|  | **-7** | **-6** | **-5** | **-4** | **-3** | **-2** | **-1** | **1** | **2** | **3** | **4** | **5** | **6** | **7** |
| --- | --- | --- | --- | --- | --- | --- | --- | --- | --- | --- | --- | --- | --- | --- |
| ***(a) P*-value of comparison of methylarginine with non-methylarginine** | | | | | | | | | | | | | | |
| **G1** | **4.16e-04** | **1.58e-07** | **1.84e-06** | **2.21e-03** | **7.11e-05** | **2.45e-04** | **1.90e-06** | **3.84e-09** | **3.72e-03** | **2.43e-05** | **3.59e-02** | **1.63e-03** | **1.22e-03** | **3.10e-04** |
| **G2** | **1.56e-02** | **2.01e-03** | **1.59e-09** | **1.00e-03** | **7.32e-02** | **1.29e-07** | **5.11e-03** | **3.07e-10** | **2.90e-07** | **8.76e-06** | **2.31e-06** | **1.91e-04** | **1.69e-06** | **6.28e-05** |
| **G3** | **1.32e-01** | **9.81e-01** | **8.35e-03** | **2.66e-05** | **7.23e-01** | **7.55e-06** | **1.87e-05** | **1.59e-06** | **4.29e-08** | **1.49e-05** | **7.26e-06** | **2.42e-02** | **1.73e-06** | **3.25e-02** |
| **G4** | **4.55e-06** | **1.27e-02** | **3.67e-08** | **5.44e-06** | **2.38e-10** | **1.05e-06** | **5.10e-05** | **3.09e-06** | **3.87e-08** | **3.03e-05** | **4.25e-05** | **9.93e-04** | **4.87e-02** | **3.26e-05** |
| **(*b*) *P*-value of comparison of methyllysine with non- methyllysine** | | | | | | | | | | | | | | |
| **G1** | **2.70e-06** | **6.64e-04** | **5.86e-04** | **4.70e-01** | **1.34e-03** | **7.95e-01** | **9.76e-04** | **1.00e-03** | **2.e-0132** | **9.41e-03** | **1.33e-01** | **6.24e-03** | **2.26e-04** | **2.49e-01** |
| **G2** | **2.10e-03** | **1.29e-04** | **4.55e-06** | **3.31e-05** | **8.46e-04** | **5.70e-05** | **8.37e-10** | **4.34e-01** | **1.78e-02** | **6.95e-05** | **6.56e-01** | **3.98e-01** | **1.44e-01** | **2.42e-01** |
| **G3** | **2.62e-08** | **7.41e-05** | **3.17e-06** | **2.07e-05** | **1.16e-07** | **5.09e-05** | **2.43e-03** | **2.81e-04** | **6.73e-05** | **7.72e-02** | **2.61e-03** | **9.50e-03** | **1.53e-01** | **2.71e-03** |
| **G4** | **4.04e-01** | **7.41e-01** | **1.11e-01** | **7.82e-03** | **4.17e-02** | **1.74e-04** | **4.53e-02** | **1.98e-06** | **2.18e-04** | **4.61e-02** | **4.82e-01** | **7.63e-03** | **5.28e-08** | **3.91e-01** |
