# Supplementary material for: PMeS: Prediction of Methylation Sites Based on Enhanced Feature Encoding Scheme
Source: PLoS One. 2012 Jun 15;7(6):e38772. doi: 10.1371/journal.pone.0038772 (PMC3376144; doi:10.1371/journal.pone.0038772)
Supplement: Table S7 — Average van der Waals volume (VDWV) of residues around methylation sites and non-methylation sites was compared via P -values on the paired Welch's t-test. (DOC) [file pone.0038772.s007.doc]

**Table S7. Average van der Waals volume (VDWV) of residues around methylation sites and non-methylation sites was compared via *P*-values on the paired Welch's t-test. There is statistical difference when *P*$\leq$0.05, or else there isn’t significantly different.**

| ***P-*value** | **-7** | **-6** | **-5** | **-4** | **-3** | **-2** | **-1** | **1** | **2** | **3** | **4** | **5** | **6** | **7** |
| --- | --- | --- | --- | --- | --- | --- | --- | --- | --- | --- | --- | --- | --- | --- |
| **Arginine** | **6.67e-02** | **3.89e-04** | **1.85e-05** | **5.53e-02** | **2.38e-09** | **7.99e-07** | **1.37e-08** | **1.18e-11** | **1.37e-04** | **5.21e-07** | **8.00e-08** | **1.49e-06** | **7.43e-02** | **1.96e-05** |
| **Lysine** | **2.09e-06** | **2.71e-06** | **1.24e-05** | **7.81e-02** | **1.27e-06** | **1.49e-09** | **5.45e-06** | **1.82e-01** | **4.60e-01** | **3.05e-01** | **4.58e-01** | **8.86e-02** | **6.49e-05** | **5.94e-06** |
