# Supplementary material for: PMeS: Prediction of Methylation Sites Based on Enhanced Feature Encoding Scheme
Source: PLoS One. 2012 Jun 15;7(6):e38772. doi: 10.1371/journal.pone.0038772 (PMC3376144; doi:10.1371/journal.pone.0038772)
Supplement: Table S8 — Average accessible surface area (ASA) of residues around methylation sites and non-methylation sites was compared via P -values on the paired Welch's t-test. (DOC) [file pone.0038772.s008.doc]

**Table S8. Average accessible surface area (ASA) of residues around methylation sites and non-methylation sites was compared via *P*-values on the paired Welch's t-test. There is statistical difference when *P*$\leq$0.05, or else there isn’t significantly different.**

| ***P*-value** | **-7** | **-6** | **-5** | **-4** | **-3** | **-2** | **-1** | **0** | **1** | **2** | **3** | **4** | **5** | **6** | **7** |
| --- | --- | --- | --- | --- | --- | --- | --- | --- | --- | --- | --- | --- | --- | --- | --- |
| **Arginine** | **3.33e-03** | **8.15e-05** | **1.04e-01** | **3.53e-02** | **3.95e-06** | **6.13e-02** | **2.73e-05** | **1.42e-03** | **5.21e-03** | **3.62e-05** | **9.35e-01** | **1.73e-03** | **1.13e-05** | **1.10e-05** | **7.94e-03** |
| **Lysine** | **6.06e-01** | **4.41e-03** | **8.78e-02** | **4.53e-03** | **1.63e-07** | **3.47e-01** | **3.06e-02** | **2.95e-01** | **8.87e-01** | **1.13e-02** | **4.03e-04** | **6.12e-02** | **2.71e-01** | **6.53e-03** | **1.85e-04** |
