# Supplementary material for: PMeS: Prediction of Methylation Sites Based on Enhanced Feature Encoding Scheme
Source: PLoS One. 2012 Jun 15;7(6):e38772. doi: 10.1371/journal.pone.0038772 (PMC3376144; doi:10.1371/journal.pone.0038772)
Supplement: Table S9 — The list of proteins containing experimental methylation sites which are located in the protein regions with known tertiary structure of protein data bank (PDB). (DOC) [file pone.0038772.s009.doc]

**Table S9. The list of proteins containing experimental methylation sites which are located in the protein regions with known tertiary structure of protein data bank (PDB). In the RVP-Net, the residue is exposed when its ASA is more than 0.16.**

| Accession | Protein name_species | Methylated  Residue | Corresponding  Region in UniProtKB | PDB ID | Corresponding methylated site  in PDB | The experimental values of ASA in methylated site | The predicted values of ASA in methylated site |
| --- | --- | --- | --- | --- | --- | --- |
| P0A7J7 | RL11_ECOLI | K40 | 15-137 | 487D|L | K40 | 0.62 | 0.624 |
| P0A7K2 | RL7_ECOLI | K82 | 48-121 | 1RQS|A | K81 | 0.64 | 0.546 |
| P68431 | H31_HUMAN | K57 | 1-136 | 2CV5|A | K56 | 0.72 | 0.509 |
|  | K65 | 1-136 | 2CV5\|A | K64 | 0.51 | 0.542 |
|  | K80 | 1-136 | 2CV5\|A | K79 | 0.18 | 0.498 |
|  | K123 | 1-136 | 2CV5\|A | K122 | 0.54 | 0.505 |
| P84229 | H32_CHICK | K80 | 1-136 | 2HIO|C | K79 | 0.32 | 0.503 |
| P10587 | MYH11_CHICK | K128 | 3-819 | 1BR1|A | K128 | 0.53 | 0.468 |
| P04637 | P53_HUMAN | K372 | 367-386 | 1JSP|A | K372 | 1.00 | 0.604 |
| P68696 | PRO1A_ACACA | K104 | 1-126 | 1ACF | K103 | 0.84 | 0.429 |
| P43320 | CRBB2_HUMAN | K42 | 1-205 | 1YTQ|A | K41 | 0.57 | 0.609 |
|  | K68 | 1-205 | 1YTQ\|A | K67 | 0.62 | 0.663 |
|  | K121 | 1-205 | 1YTQ\|A | K120 | 0.51 | 0.430 |
| P39476 | DN72_SULSO | K5 | 1-64 | 1C8C|A | K5 | 0.70 | 0.316 |
|  | K7 | 1-64 | 1C8C\|A | K7 | 0.61 | 0.352 |
|  | K61 | 1-64 | 1C8C\|A | K61 | 0.82 | 0.646 |
|  | K63 | 1-64 | 1C8C\|A | K63 | 1.00 | 0.721 |
|  | K64 | 1-64 | 1C8C\|A | K64 | 1.00 | 0.642 |
| P02994 | EF1A_YEAST | K30 | 1-458 | 1F60|A | K30 | 0.40 | 0.213 |
|  | K79 | 1-458 | 1F60\|A | K79 | 0.34 | 0.339 |
|  | K316 | 1-458 | 1IJF\|A | K316 | 0.90 | 0.397 |
|  | K390 | 1-458 | 1F60\|A | K390 | 0.78 | 0.61 |
| P62158 | CALM_HUMAN | K116 | 1-149 | 2F3Z|A | K115 | 0.80 | 0.557 |
| P07463 | CALM_PARTE | K14 | 1-149 | 1EXR|A | K13 | 0.60 | 0.394 |
|  | K116 | 1-149 | 1EXR\|A | K115 | 0.76 | 0.557 |
| P04573 | CAVP_BRALA | K96 | 82-162 | 1C7V|A | K95 | 0.55 | 0.408 |
|  | K117 | 82-162 | 1C7V\|A | K116 | 0.68 | 0.345 |
| P11442 | CLH_RAT | K245 | 1-495 | 1BPO|A | K245 | 0.55 | 0.545 |
|  | K246 | 4-360 | 1C9L\|A | K246 | 0.18 | 0.546 |
| P22498 | BGAL_SULSO | K116 | 1-489 | 1GOW|A | K116 | 0.60 | 0.447 |
|  | K135 | 1-489 | 1GOW\|A | K135 | 0.51 | 0.687 |
|  | K273 | 1-489 | 1GOW\|A | K273 | 0.80 | 0.545 |
|  | K311 | 1-489 | 1GOW\|A | K311 | 0.46 | 0.493 |
|  | K332 | 1-489 | 1GOW\|A | K332 | 0.58 | 0.557 |
| P39462 | ADH_SULSO | K11 | 1-347 | 1JVB|A | K11 | 0.16 | 0.487 |
|  | K213 | 1-347 | 1JVB\|A | K213 | 0.62 | 0.502 |
| P0CX53 | RL12_YEAST | R67 | 13-143 | 1S1I|K | R67 | 0.78 | 0.159 |
| P42224 | STAT1_HUMAN | R31 | 1-683 | 1YVL|A | R31 | 0.01 | 0.130 |
| P68431 | H31_HUMAN | R129 | 1-136 | 2CV5|A | R128 | 0.11 | 0.365 |
|  | R130 | 1-136 | 2CV5\|A | R129 | 0.42 | 0.442 |
| P53674 | CRBB1_HUMAN | R230 | 43-252 | 1OKI|A | R230 | 0.01 | 0.406 |
|  | R231 | 43-252 | 1OKI\|A | R231 | 0.10 | 0.391 |
| P68433 | H31_MOUSE | R9 | 1-17 | 1GUW|B | R8 | 0.31 | 0.365 |
