# Supplementary material for: PMeS: Prediction of Methylation Sites Based on Enhanced Feature Encoding Scheme
Source: PLoS One. 2012 Jun 15;7(6):e38772. doi: 10.1371/journal.pone.0038772 (PMC3376144; doi:10.1371/journal.pone.0038772)
Supplement: Table S10 — The predictive performance of model trained with different features was compared via P -values on the paired Welch's t-test. (DOC) [file pone.0038772.s010.doc]

**Table S10. The predictive performance of models trained with different features was compared via *P*-values on the paired Welch's t-test.** For the entry at row *i*, column *j* of the table, there is statistical difference when *P*$\leq$0.05, or else there isn’t significantly different. The ratio between positive and negative samples was 1:3. SPC: sparse property coding; PWAA: position weight amino acids composition; ASA: **s**olvent accessible surface area; VDWV: Van der Waals volume; SPA: SPC+PWAA+ASA; SPV: SPC+PWAA+VDWV; SPAV: SPC+PWAA+ASA+VDWV.

|  | **SPC** | **PWAA** | **ASA** | **VDWV** | **SPC+PWAA** | **SPC+ASA** | **PWAA+ASA** | **SPA** | **SPV** | **SPAV** |
| --- | --- | --- | --- | --- | --- | --- | --- | --- | --- | --- |
| *(a) P*-value of sensitivity comparisons of methylarginine | | | | | | | | | | |
| **SPC** | **1.00** | **3.67e-06** | **7.28e-09** | **1.60e-07** | **7.33e-02** | **5.66e-03** | **7.88e-02** | **6.92e-04** | **1.41e-01** | **3.77e-04** |
| **PWAA** |  | **1.00** | **6.12e-10** | **4.53e-06** | **1.34e-05** | **4.11e-05** | **9.05e-04** | **6.28e-08** | **3.67e-03** | **6.17e-06** |
| **ASA** |  |  | **1.00** | **2.57e-03** | **1.14e-07** | **6.96e-09** | **8.03e-07** | **6.23e-10** | **1.54e-04** | **3.42e-07** |
| **VDWV** |  |  |  | **1.00** | **6.91e-07** | **4.36e-07** | **7.51e-06** | **1.24e-08** | **3.55e-04** | **9.01e-07** |
| **SPC+PWAA** |  |  |  |  | **1.00** | **1.38e-03** | **9.42e-03** | **1.12e-01** | **4.35e-01** | **3.31e-03** |
| **SPC+ASA** |  |  |  |  |  | **1.00** | **5.57e-01** | **9.91e-06** | **3.18e-02** | **6.02e-05** |
| **PWAA+ASA** |  |  |  |  |  |  | **1.00** | **2.95e-04** | **5.01e-02** | **1.68e-04** |
| **SPA** |  |  |  |  |  |  |  | **1.00** | **8.61e-01** | **9.55e-03** |
| **SPV** |  |  |  |  |  |  |  |  | **1.00** | **4.90e-02** |
| **SPAV** |  |  |  |  |  |  |  |  |  | **1.00** |
| *(b) P*-value of sensitivity comparisons of methyllysine | | | | | | | | | | |
| **SPC** | **1.00** | **7.65e-04** | **3.86e-04** | **2.21e-03** | **1.76e-03** | **1.14e-01** | **4.19-01** | **2.47e-04** | **1.30e-02** | **2.95e-06** |
| **PWAA** |  | **1.00** | **3.96e-03** | **6.12e-02** | **3.36e-05** | **6.25e-03** | **2.53e-02** | **2.44e-05** | **2.08e-03** | **3.69e-07** |
| **ASA** |  |  | **1.00** | **1.19e-01** | **4.46e-05** | **5.58e-04** | **1.31e-03** | **1.82e-05** | **3.29e-04** | **1.79e-06** |
| **VDWV** |  |  |  | **1.00** | **1.18e-04** | **2.46e-03** | **7.11e-03** | **3.89e-05** | **1.00e-03** | **2.35e-06** |
| **SPC+PWAA** |  |  |  |  | **1.00** | **4.17e-01** | **1.24e-01** | **2.48e-02** | **2.38e-01** | **2.34e-04** |
| **SPC+ASA** |  |  |  |  |  | **1.00** | **5.12e-01** | **2.17e-02** | **1.35e-01** | **8.09e-04** |
| **PWAA+ASA** |  |  |  |  |  |  | **1.00** | **7.01e-03** | **5.84e-02** | **3.02e-04** |
| **SPA** |  |  |  |  |  |  |  | **1.00** | **6.87e-01** | **4.77e-02** |
| **SPV** |  |  |  |  |  |  |  |  | **1.00** | **4.71e-02** |
| **SPAV** |  |  |  |  |  |  |  |  |  | **1.00** |
