# Supplementary material for: PMeS: Prediction of Methylation Sites Based on Enhanced Feature Encoding Scheme
Source: PLoS One. 2012 Jun 15;7(6):e38772. doi: 10.1371/journal.pone.0038772 (PMC3376144; doi:10.1371/journal.pone.0038772)
Supplement: Table S11 — The performance of models trained with different window sizes for methylarginine. (DOC) [file pone.0038772.s011.doc]

**Table S11. The performance of models trained with different window sizes for methylarginine. The corresponding measurement was represented as the average value ± standard deviation. The ratio between positive and negative samples was 1:3 and training feature was SPC+PWAA+ASA+VDWV.**

| Window size | Sensitivity | Specificity | Accuracy | MCC |
| --- | --- | --- | --- | --- |
| -4~R~+4 | 66.03±1.58 | 91.14±0.73 | 84.86±0.44 | 58.69±1.14 |
| -5~R~+5 | 74.31±4.63 | 92.45±0.68 | 87.92±1.02 | 67.51±4.20 |
| -6~R~+6 | 68.73±2.41 | 90.95±0.77 | 85.39±0.82 | 60.54±2.29 |
| -7~R~+7 | 80.73±1.58 | 92.28±1.24 | 89.39±1.35 | 72.45±1.73 |
| -8~R~+8 | 70.82±2.42 | 90.55±1.29 | 85.62±0.73 | 61.59±1.67 |
| -9~R~+9 | 70.76±3.36 | 91.29±2.21 | 86.16±1.02 | 62.86±1.97 |
