# Supplementary material for: PMeS: Prediction of Methylation Sites Based on Enhanced Feature Encoding Scheme
Source: PLoS One. 2012 Jun 15;7(6):e38772. doi: 10.1371/journal.pone.0038772 (PMC3376144; doi:10.1371/journal.pone.0038772)
Supplement: Table S12 — The performance of models trained with different window sizes for methyllysine. (DOC) [file pone.0038772.s012.doc]

**Table S12. The performance of models trained with different window sizes for methyllysine. The corresponding measurement was represented as the average value ± standard deviation. The ratio between positive and negative samples was 1:3 and training feature was SPC+PWAA+ASA+VDW.**

| Window size | Sensitivity | Specificity | Accuracy | MCC |
| --- | --- | --- | --- | --- |
| -4~K~+4 | 67.77±1.98 | 99.46±0.35 | 91.56±0.48 | 76.91±1.30 |
| -5~K~+5 | 68.50±2.85 | 98.94±0.31 | 91.37±0.62 | 76.17±1.75 |
| -6~K~+6 | 68.56±3.78 | 99.05±0.31 | 91.46±0.40 | 76.42±2.67 |
| -7~K~+7 | 73.56±2.08 | 99.11±0.39 | 92.75±0.25 | 80.15±0.64 |
| -8~K~+8 | 73.25±2.26 | 98.92±0.22 | 92.54±0.63 | 79.48±1.81 |
| -9~K~+9 | 66.69±1.41 | 99.48±0.23 | 91.32±0.22 | 76.20±0.60 |
