# Supplementary material for: PMeS: Prediction of Methylation Sites Based on Enhanced Feature Encoding Scheme
Source: PLoS One. 2012 Jun 15;7(6):e38772. doi: 10.1371/journal.pone.0038772 (PMC3376144; doi:10.1371/journal.pone.0038772)
Supplement: Table S13 — The predictive result of models with different window sizes was compared via P -values on the paired Welch's t-test. (DOC) [file pone.0038772.s013.doc]

**Table S13. The predictive result of models with different window sizes was compared via *P*-values on the paired Welch's t-test. The ratio between positive and negative samples was 1:3 and training feature was SPC+PWAA+ASA+VDW.**

| **Window**  **Size** | **9** | **11** | **13** | **15** | **17** | **19** |
| --- | --- | --- | --- | --- | --- | --- |
|  | ***(a) P*-value of sensitivity comparisons of methylarginine** | | | | | |
| **9** | 1.00 | 6.71e-02 | 6.93e-02 | 8.46e-05 | 6.02e-03 | 2.14e-02 |
| **11** |  | 1.00 | 2.00e-01 | 1.73e-01 | 4.08e-01 | 4.15e-01 |
| **13** |  |  | 1.00 | 5.64e-04 | 2.10e-01 | 3.05e-01 |
| **15** |  |  |  | 1.00 | 1.87e-03 | 3.29e-03 |
| **17** |  |  |  |  | 1.00 | 9.76e-01 |
| **19** |  |  |  |  |  | 1.00 |
| ***(b) P*-value of sensitivity comparisons of methyllysine** | | | | | | |
| **9** | 1.00 | 7.23e-01 | 8.44e-01 | 2.36e-03 | 4.22e-03 | 2.83e-01 |
| **11** |  | 1.00 | 9.85e-01 | 1.25e-02 | 1.93e-02 | 2.37e-01 |
| **13** |  |  | 1.00 | 1.44e-01 | 1.71e-01 | 5.51e-01 |
| **15** |  |  |  | 1.00 | 8.26e-01 | 2.82e-04 |
| **17** |  |  |  |  | 1.00 | 5.63e-04 |
| **19** |  |  |  |  |  | 1.00 |
