# Supplementary material for: PMeS: Prediction of Methylation Sites Based on Enhanced Feature Encoding Scheme
Source: PLoS One. 2012 Jun 15;7(6):e38772. doi: 10.1371/journal.pone.0038772 (PMC3376144; doi:10.1371/journal.pone.0038772)
Supplement: Table S14 — The performance of models trained with different ratio of positive to negative samples for methylarginine. (DOC) [file pone.0038772.s014.doc]

**Table S14. The performance of models trained with different ratio of positive to negative samples for methylarginine.** The corresponding measurement was represented as the average value ± standard deviation**.** The window size was 15 and training feature was SPC+PWAA+ASA+VDW.

| The ratio of positive to negative samples | The predictive performance (%) | | | |
| --- | --- | --- | --- | --- |
|  | Sn | Sp | Acc | MCC |
| 1:1 | 92.45±1.43 | 93.18±2.66 | 92.82±1.79 | 85.69±3.78 |
| 1:2 | 82.82±1.48 | 93.01±1.27 | 89.62±0.95 | 76.49±2.06 |
| 1:3 | 80.73±1.58 | 92.28±1.24 | 89.39±1.35 | 72.45±1.73 |
| 1:4 | 73.75±0.37 | 96.17±1.83 | 91.69±1.72 | 73.54±3.32 |
| 1:5 | 59.61±1.24 | 99.61±0.08 | 92.94±0.25 | 72.71±1.09 |
| 1:6 | 51.38±1.54 | 99.48±0.16 | 92.61±0.32 | 66.44±1.69 |
| 1:7 | 46.54±1.40 | 99.68±0.06 | 93.03±0.19 | 63.89±1.18 |
| 1:8 | 44.06±0.62 | 99.68±0.09 | 93.50±0.10 | 62.05±0.70 |
