# Supplementary material for: PMeS: Prediction of Methylation Sites Based on Enhanced Feature Encoding Scheme
Source: PLoS One. 2012 Jun 15;7(6):e38772. doi: 10.1371/journal.pone.0038772 (PMC3376144; doi:10.1371/journal.pone.0038772)
Supplement: Table S15 — The performance of models trained with different ratio of positive to negative samples for methyllysine. (DOC) [file pone.0038772.s015.doc]

**Table S15. The performance of models trained with different ratio of positive to negative samples for methyllysine.** The corresponding measurement was represented as the average value ± standard deviation**.** The window size was 15 and training feature was SPC+PWAA+ASA+VDW.

| The ratio of positive to negative samples | The predictive performance (%) | | | |
| --- | --- | --- | --- | --- |
|  | Sn | Sp | Acc | MCC |
| 1:1 | 84.38±1.06 | 93.94±0.87 | 89.16±0.66 | 78.68±1.31 |
| 1:2 | 77.44±2.44 | 98.66±0.44 | 91.58±0.60 | 81.09±1.68 |
| 1:3 | 73.56±2.08 | 99.11±0.39 | 92.75±0.25 | 80.15±0.64 |
| 1:4 | 65.13±1.92 | 99.39±0.31 | 92.57±0.36 | 75.51±1.28 |
| 1:5 | 58.56±1.00 | 99.61±0.17 | 92.79±0.13 | 72.02±0.59 |
| 1:6 | 44.31±1.92 | 99.71±0.19 | 91.84±0.27 | 62.15±1.53 |
| 1:7 | 47.81±1.55 | 99.79±0.13 | 93.32±0.24 | 65.47±1.46 |
| 1:8 | 44.94±0.87 | 99.81±0.08 | 93.73±0.05 | 63.54±0.36 |
