# Supplementary material for: PMeS: Prediction of Methylation Sites Based on Enhanced Feature Encoding Scheme
Source: PLoS One. 2012 Jun 15;7(6):e38772. doi: 10.1371/journal.pone.0038772 (PMC3376144; doi:10.1371/journal.pone.0038772)
Supplement: Table S16 — The predictive result of models with different ratios of positive to negative samples was compared via P -values on the paired Welch's t-test. (DOC) [file pone.0038772.s016.doc]

**Table S16. The predictive result of models with different ratios of positive to negative samples was compared via *P*-values on the paired Welch's t-test. The window size was 15 and training feature was SPC+PWAA+ASA+VDWV.**

|  | **1:1** | **1:2** | **1:3** | **1:4** | **1:5** | **1:6** | **1:7** | **1:8** |
| --- | --- | --- | --- | --- | --- | --- | --- | --- |
| *(a) P*-value of MCC comparisons of methylarginine | | | | | | | | |
| **1:1** | **1.00** | **7.61e-03** | **4.46e-03** | **1.42e-02** | **7.85e-04** | **6.49e-05** | **2.22e-05** | **1.10e-05** |
| **1:2** |  | **1.00** | **1.49e-01** | **3.80e-01** | **6.81e-03** | **3.00e-05** | **2.33e-06** | **4.22e-07** |
| **1:3** |  |  | **1.00** | **7.84e-01** | **9.15e-01** | **4.12e-02** | **7.46e-03** | **2.36e-03** |
| **1:4** |  |  |  | **1.00** | **7.95e-01** | **5.32e-02** | **1.39e-02** | **5.55e-03** |
| **1:5** |  |  |  |  | **1.00** | **1.16e-04** | **1.78e-06** | **7.85e-08** |
| **1:6** |  |  |  |  |  | **1.00** | **2.38e-02** | **6.77e-04** |
| **1:7** |  |  |  |  |  |  | **1.00** | **1.79e-02** |
| **1:8** |  |  |  |  |  |  |  | **1.00** |
| *(b) P*-value of MCC comparisons of methyllysine | | | | | | | | |
| **1:1** | **1.00** | **1.85e-02** | **5.43e-02** | **4.77e-03** | **6.32e-06** | **7.97e-08** | **3.72e-07** | **7.00e-09** |
| **1:2** |  | **1.00** | **1.79e-01** | **1.27e-04** | **5.21e-07** | **2.55e-08** | **9.36e-08** | **1.85e-09** |
| **1:3** |  |  | **1.00** | **9.02e-05** | **2.83e-08** | **8.95e-09** | **3.28e-08** | **2.53e-11** |
| **1:4** |  |  |  | **1.00** | **5.44e-04** | **3.93e-07** | **2.86e-06** | **3.88e-08** |
| **1:5** |  |  |  |  | **1.00** | **8.92e-07** | **1.45e-05** | **3.13e-09** |
| **1:6** |  |  |  |  |  | **1.00** | **8.08e-03** | **8.41e-02** |
| **1:7** |  |  |  |  |  |  | **1.00** | **2.09e-02** |
| **1:8** |  |  |  |  |  |  |  | **1.00** |
