# Supplementary material for: PMeS: Prediction of Methylation Sites Based on Enhanced Feature Encoding Scheme
Source: PLoS One. 2012 Jun 15;7(6):e38772. doi: 10.1371/journal.pone.0038772 (PMC3376144; doi:10.1371/journal.pone.0038772)
Supplement: Table S17 — The performance of the methylarginine model based on self-consistency, K-fold (4-, 6-, 8- and 10-fold) cross-validation and leave-one-out validation. (DOC) [file pone.0038772.s017.doc]

**Table S17. The performance of the methylarginine model based on self-consistency, K-fold (4-, 6-, 8- and 10-fold) cross-validation and leave-one-out validation.** The corresponding measurement was represented as the average value ± standard deviation**.** The window size was 15, the ratio between positive and negative samples was 1:1 and training feature was SPC+PWAA+ASA+VDW.

| Cross Validation | The predictive performance (%) | | | |
| --- | --- | --- | --- | --- |
|  | Sn | Sp | Acc | MCC |
| Self | 93.07±2.41 | 93.52±1.42 | 93.30±1.89 | 86.60±3.79 |
| 4 | 92.85±1.99 | 92.06±1.51 | 92.45±1.42 | 84.92±2.84 |
| 6 | 93.30±2.13 | 92.56±2.49 | 92.93±2.12 | 85.88±4.24 |
| 8 | 93.41±2.19 | 92.73±2.85 | 93.07±2.46 | 86.15±4.92 |
| 10 | 92.45±1.43 | 93.18±2.66 | 92.82±1.79 | 85.69±3.78 |
| LOOV | 93.30±2.20 | 92.68±2.42 | 92.99±2.08 | 85.99±4.14 |
