# Supplementary material for: PMeS: Prediction of Methylation Sites Based on Enhanced Feature Encoding Scheme
Source: PLoS One. 2012 Jun 15;7(6):e38772. doi: 10.1371/journal.pone.0038772 (PMC3376144; doi:10.1371/journal.pone.0038772)
Supplement: Table S18 — MCC of methylarginine model by different cross-validation was compared via P -values on the paired Welch's t-test. (DOC) [file pone.0038772.s018.doc]

**Table S18. MCC of methylarginine model by different cross-validation was compared via *P*-values on the paired Welch's t-test. The window size was 15, the ratio between positive and negative samples was 1:1 and training feature was SPC+PWAA+ASA+VDWV. Self: self-consistency; CV: cross-validation; LOOV: leave-one-out validation.**

| ***P*-value** | **Self** | **4-CV** | **6-CV** | **8-CV** | **10-CV** | **LOOV** |
| --- | --- | --- | --- | --- | --- | --- |
| **Self** | 1.00 | 6.29e-01 | 8.36e-01 | 8.94e-01 | 7.97e-01 | 8.67e-01 |
| **4-CV** |  | 1.00 | 7.79e-01 | 7.09e-01 | 8.24e-01 | 7.66e-01 |
| **6-CV** |  |  | 1.00 | 9.34e-01 | 9.57e-01 | 9.75e-01 |
| **8-CV** |  |  |  | 1.00 | 8.91e-01 | 9.63e-01 |
| **10-CV** |  |  |  |  | 1.00 | 9.35e-01 |
| **LOOV** |  |  |  |  |  | 1.00 |
