# Supplementary material for: PMeS: Prediction of Methylation Sites Based on Enhanced Feature Encoding Scheme
Source: PLoS One. 2012 Jun 15;7(6):e38772. doi: 10.1371/journal.pone.0038772 (PMC3376144; doi:10.1371/journal.pone.0038772)
Supplement: Table S19 — We collected 46 experimentally identified methyllysine sites in 39 unique proteins from the scientific literature (PubMed). (DOC) [file pone.0038772.s019.doc]

**Table S19.** **We collected 46 experimentally identified methyllysine sites in 39 unique proteins from the scientific literature (PubMed).** PMID: the primary references for the experimentally verified methyllysine sites.

| Accession number | Residue position | Species | PMID |
| --- | --- | --- | --- |
| P59230 | K90 | ARATH | 17934214 |
| Q8VZB9 | K90 | ARATH | 17934214 |
| P38328 | K121 | YEAST | 20137074 |
| P00830 | K196 | YEAST | 20137074 |
| Q04660 | K577,581 | YEAST | 20137074 |
| P29453 | K15,43,241 | YEAST | 20137074 |
| P32565 | K376 | YEAST | 20137074 |
| P05756 | K140 | YEAST | 20137074 |
| P02407 | K59 | YEAST | 20137074 |
| Q12464 | K412 | YEAST | 20137074 |
| P17883 | K1088 | YEAST | 20137074 |
| P40018 | K138,145 | YEAST | 20137074 |
| P22082 | K1028 | YEAST | 20137074 |
| P38249 | K192 | YEAST | 20137074 |
| P28274 | K28 | YEAST | 20137074 |
| P25386 | K119 | YEAST | 20137074 |
| P53254 | K1158 | YEAST | 20137074 |
| Q12220 | K350 | YEAST | 20137074 |
| P32660 | K541 | YEAST | 20137074 |
| Q06287 | K147 | YEAST | 20137074 |
| Q03532 | K444 | YEAST | 20137074 |
| P38144 | K14 | YEAST | 20137074 |
| P41940 | K299 | YEAST | 20137074 |
| P22354 | K104 | YEAST | 20137074 |
| P32497 | K514 | YEAST | 20137074 |
| P39744 | K384 | YEAST | 20137074 |
| P38121 | K84 | YEAST | 20137074 |
| P53131 | K662 | YEAST | 20137074 |
| P22138 | K513 | YEAST | 20137074 |
| P26784 | K148 | YEAST | 20137074 |
| P0CX43 | K207 | YEAST | 20137074 |
| P0CX23 | K47 | YEAST | 20137074 |
| P36528 | K70 | YEAST | 20137074 |
| Q06839 | K249 | YEAST | 20137074 |
| A4YEA2 | K53 | METS | 19850496 |
| Q701L7 | K137,354 | HUMAN | 16446289 |
| B2R4R0 | K32,60 | HUMAN | 16446289 |
| A8K872 | K377 | HUMAN | 16446289 |
| B2R4S9 | K47,109 | HUMAN | 16446289 |
