# Supplementary material for: PMeS: Prediction of Methylation Sites Based on Enhanced Feature Encoding Scheme
Source: PLoS One. 2012 Jun 15;7(6):e38772. doi: 10.1371/journal.pone.0038772 (PMC3376144; doi:10.1371/journal.pone.0038772)
Supplement: Table S20 — We collected 27 experimentally identified methylarginine sites in 24 unique proteins from the scientific literature (PubMed). (DOC) [file pone.0038772.s020.doc]

**Table S20.** **We collected 27 experimentally identified methylarginine sites in 24 unique proteins from the scientific literature (PubMed).** PMID: the primary references for the experimentally verified methylarginine sites.

| Accession number | Residue position | Species | PMID |
| --- | --- | --- | --- |
| P32458 | R35 | YEAST | 20137074 |
| P34216 | R252 | YEAST | 20137074 |
| P38631 | R946,952,962,1527 | YEAST | 20137074 |
| P46655 | R371 | YEAST | 20137074 |
| P50095 | R168 | YEAST | 20137074 |
| Q08963 | R141 | YEAST | 20137074 |
| P53742 | R336 | YEAST | 20137074 |
| P25655 | R256 | YEAST | 20137074 |
| P06103 | R572 | YEAST | 20137074 |
| P53037 | R252 | YEAST | 20137074 |
| P00549 | R216 | YEAST | 20137074 |
| P08518 | R496 | YEAST | 20137074 |
| P07279 | R105 | YEAST | 20137074 |
| P05736 | R21 | YEAST | 20137074 |
| Q12213 | R218 | YEAST | 20137074 |
| P26781 | R67 | YEAST | 20137074 |
| P53236 | R454 | YEAST | 20137074 |
| P38781 | R692 | YEAST | 20137074 |
| P11484 | R513 | YEAST | 20137074 |
| P00359 | R11 | YEAST | 20137074 |
| P32807 | R549 | YEAST | 20137074 |
| P02557 | R318 | YEAST | 20137074 |
| P39904 | R224 | YEAST | 20137074 |
| Q05022 | R215 | YEAST | 20137074 |
