# Supplementary material for: PMeS: Prediction of Methylation Sites Based on Enhanced Feature Encoding Scheme
Source: PLoS One. 2012 Jun 15;7(6):e38772. doi: 10.1371/journal.pone.0038772 (PMC3376144; doi:10.1371/journal.pone.0038772)
Supplement: Table S21 — Statistical comparison of training test with independent test based on the paired Welch's t-test. (DOC) [file pone.0038772.s021.doc]

**Table S21. Statistical** **comparison of training test with independent test based on the paired Welch's t-test.** The window size was 15, the ratio between positive and negative samples was 1:1 and training feature was SPC+PWAA+ASA+VDWV.

| ***P*-value** | **Sn** | **Sp** | **Acc** | **Mcc** |
| --- | --- | --- | --- | --- |
| **Arginine** | 9.88e-04 | 1.12e-01 | 1.25e-01 | 1.67e-01 |
| **Lysine** | 3.98e-05 | 2.40e-03 | 1.11e-04 | 5.50e-03 |
