# Supplementary material for: PMeS: Prediction of Methylation Sites Based on Enhanced Feature Encoding Scheme
Source: PLoS One. 2012 Jun 15;7(6):e38772. doi: 10.1371/journal.pone.0038772 (PMC3376144; doi:10.1371/journal.pone.0038772)
Supplement: Table S22 — Statistical comparison of PMeS with MASA on the dataset adopted in MASA method. (DOC) [file pone.0038772.s022.doc]

**Table S22. Statistical** **comparison of PMeS with MASA on the dataset adopted in MASA method.** Performance of the method was compared via *P*-values on Welch's t-test.

| ***P*-value** | **Sn** | **Sp** | **Acc** | **Mcc** |
| --- | --- | --- | --- | --- |
| **Arginine** | 3.52e-02 | 2.21e-01 | 2.91e-02 | 2.78e-02 |
| **Lysine** | 4.18e-04 | 2.15e-09 | 6.35e-07 | 4.20e-08 |
